# Transcription factor UBF depletion in mouse cells results in downregulation of both downstream and upstream elements of the rRNA transcription network

**DOI:** 10.1016/j.jbc.2023.105203

**Published:** 2023-09-01

**Authors:** Andria Theophanous, Andri Christodoulou, Charalambia Mattheou, Dany S. Sibai, Tom Moss, Niovi Santama

**Affiliations:** 1 Department of Biological Sciences, University of Cyprus, Nicosia, Cyprus; 2 Laboratory of Growth and Development, St-Patrick Research Group in Basic Oncology, Cancer Division of the Quebec University Hospital Research Centre, Quebec, Canada; 3 Department of Molecular Biology, Medical Biochemistry and Pathology, Faculty of Medicine, Laval University, Quebec, Canada

**Keywords:** RNA pol1, rRNA, C-MYC, cellular growth, transcriptional regulation

## Abstract

Transcription/processing of the ribosomal RNA (rRNA) precursor, as part of ribosome biosynthesis, is intensively studied and characterized in eukaryotic cells. Here, we constructed shRNA-based mouse cell lines partially silenced for the Upstream Binding Factor UBF, the master regulator of rRNA transcription and organizer of open rDNA chromatin. Full *Ubf* silencing *in vivo* is not viable, and these new tools allow further characterization of rRNA transcription and its coordination with cellular signaling. shUBF cells display cell cycle G1 delay and reduced 47S rRNA precursor and 28S rRNA at baseline and serum-challenged conditions. Growth-related mTOR signaling is downregulated with the fractions of active phospho-S6 Kinase and pEIF4E translation initiation factor reduced, similar to phosphorylated cell cycle regulator retinoblastoma, pRB, positive regulator of UBF availability/rRNA transcription. Additionally, we find transcription-competent pUBF (Ser484) severely restricted and its interacting initiation factor RRN3 reduced and responsive to extracellular cues. Furthermore, fractional UBF occupancy on the rDNA unit is decreased in shUBF, and expression of major factors involved in different aspects of rRNA transcription is severely downregulated by UBF depletion. Finally, we observe reduced RNA Pol1 occupancy over rDNA promoter sequences and identified unexpected regulation of RNA Pol1 expression, relative to serum availability and under UBF silencing, suggesting that regulation of rRNA transcription may not be restricted to modulation of Pol1 promoter binding/elongation rate. Overall, this work reveals that UBF depletion has a critical downstream and upstream impact on the whole network orchestrating rRNA transcription in mammalian cells.

The nucleolus is the site of transcription of the ribosomal RNA (rRNA) precursor from the 150 to 220, or more, tandem rDNA gene copies, present in megabase arrays distributed over 5 different chromosomes in the mouse genome (1), and of ensuing rRNA processing and modification, and assembly of the ribosomal subunits. rRNA synthesis is thus essential for protein synthesis and one of the most complex and energy-consuming cellular processes and therefore the subject of intensive study in eukaryotic cells.

Such studies have highlighted the important role in rRNA transcription of UBF (Upstream Binding Factor), a conserved “architectural” transcription factor with several DNA binding domains, termed “HMG boxes” due to their similarity to High Mobility Group chromosomal proteins. UBF orchestrates the assembly of the transcription pre-initiation complex (PIC) of RNA polymerase I (Pol1, also termed POLR1 or RPI) on the promoter, and the establishment of active or potentially active rDNA chromatin conformation ((2, 3); reviewed by (4)).

UBF can bind throughout the entire coding sequence of the rDNA gene as well as at the upstream enhancer repeats, enhancer-associated “spacer promoter,” and the 47S-pre-rRNA promoter (2, 5, 6). Promoter recognition and PIC assembly depend on synergistic co-operative interactions between UBF and the multi-component SL1 transcription factor complex that is composed of the TATA-binding protein (TBP) and TAF factors A-D (3, 7). The recruitment of the RNA polymerase 1 holoenzyme, composed of 14 subunits in mice, to the UBF/SL1 PIC is mediated by the factor RRN3 *via* Pol1 subunits PAF53 and PAF49 (8, 9, 10, 11, 12) and facilitated by interactions with UBF (13, 14). Following transcription initiation, the polymerase is released from the PIC, transitioning into the early elongation complex from which RRN3 is released stochastically. Finally, transcription termination occurs when the elongating polymerase reaches the transcription termination factor (TTF1) bound at the 3′ T1-T10 terminator elements at the downstream gene boundary, causing Pol1 arrest (15, 16).

The role of UBF phosphorylation in the assembly of PIC and rDNA transcription is considered to be critical. UBF harbors a large number of Ser/Thr phosphorylation sites that are targets of signaling pathways (MAPK/PI3K/mTOR/JNK) and mediated by several kinases including cdk/cyclins, CKI, CKII, CDC2, GSK, and PI3K [W1]. Importantly, UBF binding to rDNA enhancer/promoter regulatory sequences or its protein interactions with the transcription machinery, including SL1 and Pol1, are dependent on those phosphorylations (17, 18, 19, 20, 21, 22, 23). UBF is bound tightly to rDNA repeat units on condensed chromosomes in mitosis; post-mitosis, it is subject to phosphorylation of S484 by CDK4/cyclin D at G1 and CDK2/cyclin E at S phase, and by phosphorylation of S388 by CDK2/cyclin A at G2, with rRNA transcriptional rate peaking at S and G2 ((23, 24); reviewed by (25)).

Regarding the impact of UBF on rDNA chromatin conformation, UBF is thought to bind to ∼140 bp of DNA as a dimer, inducing a single DNA loop in a structure termed the «enhancesome» (26). UBF can displace linker histone H1 (27, 28). UBF thus maintains rDNA in a transcription-competent, decondensed, and nucleosome-free, “open” state. In this way, UBF mediates the formation of active or potentially active rDNA chromatin and facilitates Pol1 PIC formation. Neither RRN3 nor Pol 1 transcription is suggested to be required to maintain this potentially active state of the rRNA genes (28). Μore recent work indicates that the potentially active state is not constitutive and is gradually lost after transcription inactivation (3, 29).

There are two alternatively spliced isoforms in the mouse, UBF1 (94 kDa) and UBF2 (90 kDa), which are otherwise identical except for 37 aa in HMG box 2 of UBF2 removed by alternative splicing (30). Most studies have focused on UBF1 since UBF2 does not appear associated with Pol1 PIC formation *in vitro* or *in vivo* (31, 32, 33). UBF2 has not received equal attention although UBF2 is conserved in mammals, is of similar or higher cellular concentration to UBF1, and is also subject to the same phosphorylation events (33, 34). Both isoforms are also implicated in Pol II-driven transcription of certain genes (35, 36).

Overall, it is clear that UBF has a multi-pronged involvement and significance in the regulation of rRNA transcription. Despite great advances with the identification of many of the key factors, their protein interactions, and mechanisms involved, important questions remain regarding the structure of the PIC, the mechanisms of its assembly, the modulation of Pol1 activity, and the co-ordination of rRNA transcriptional regulation with cell signaling and cell growth, and are thus the focus of on-going research.

In this work, we wish to investigate further the various roles of UBF by studying the effects of a reduction in its expression. To achieve this, we have constructed cell lines that are partially silenced for UBF *via* shRNA-mediated lentiviral transduction. These cells maintain a haploid-like, viable level of UBF expression, and so can provide information that was inaccessible in studies with *Ubf* gene deletion which is embryonically lethal (6, 37). Thus, this new tool gives the opportunity to study rRNA transcription under prolonged, viable, reduced UBF levels that may reveal longer-term adaptive, rather than acute responses. Partial silencing and partial phenotypes also allow probing of the interconnections and co-ordination of both extrinsic signaling and intrinsic protein networks to the regulation of rRNA synthesis. In this work, we find that UBF downregulation not only significantly impacts rRNA transcription but it results in the downregulation of factors involved in initiation and termination, chromatin structure, and cell-cycle-dependent phosphorylation. Upstream *C-myc* gene expression as well as upstream mTOR growth signaling are also negatively impacted by UBF depletion, and, surprisingly, gene expression of RNA Pol1 is also downregulated. Our new findings provide novel insight into the co-ordinate regulation of the network underlying the multifaceted regulation of rRNA transcription.

## Results

### Construction and characterization of shUBF cell lines with decreased pre-rRNA/rRNA expression

**Figure 1:**
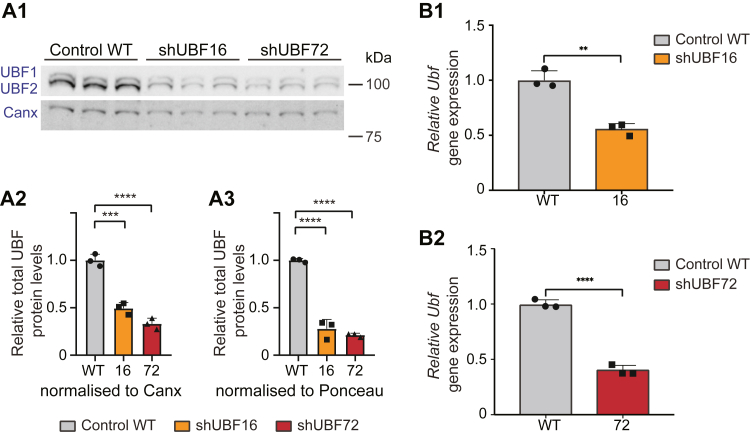
**Generation and validation of clones stably silenced for *Ubf*.***A1*, comparison by WB of UBF protein levels (UBF1 and UBF2) in WT, shUBF clone 16 and 72 (*upper panel*), and calnexin levels for normalization (*lower panel*) in samples from three independent experiments. *A2* and *A3*, corresponding quantification of the three independent experiments shown in *A1*, depicting the average value of total UBF protein levels normalized to either calnexin (*A2*), or total protein (*A3*; as visualized by Ponceau staining of the immunoblot membrane shown in Fig. S1*A*), and expressed relative to the WT control value (set at 1) ± standard deviation (SD). The analysis with both normalization methods shows a marked reduction in UBF protein levels well below 50% of WT levels in both shUBF16 and 72. Statistical significance of differences was assessed by one-way ANOVA with Dunnett’s test. *B1* and *B2*, quantification of relative *Ubf* mRNA levels by qRT-PCR, depicting respectively, a normalized average knockdown in shUBF16 (*B1*) or shUBF72 (*B2*), expressed as a fraction of the normalized average WT control values. Error bars correspond to the SD of three independent experiments. *B2m*+*PumI* housekeeping gene mRNA levels were used for sample normalization. Statistical significance of differences was assessed by Welch’s *t* test.

To initiate our investigation, we used lentiviral vectors harboring shRNAs that targeted mouse *Ubf* and transduced the NIH 3T3 fibroblast cell line to generate cells constitutively expressing the *Ubf-*silencing shRNAs. A total of 78 puromycin-resistant single clones were hand-picked, expanded, and subjected to initial screening by Western blotting (WB), using an anti-UBF antibody, to identify those with reduced UBF protein, as compared with wild-type levels (WT) in the maternal NIH 3T3 line. Two clones, named shUBF16 and shUBF72, which displayed the lowest UBF protein levels, as normalized to endogenous calnexin in this initial screen, were further analyzed in three independent experiments; these confirmed a robust reduction of total UBF to below 50% of WT in both clones (Fig. 1*A1*). In particular, UBF protein levels in shUBF16 were 0.49 ± 0.06 (relative ratio to WT control set to 1 ± SD) when normalized to same-sample calnexin levels (Fig. 1*A2*), or 0.28 ± 0.09 when normalized to total protein, as determined by whole-sample Ponceau staining of the WB membrane (Figs. 1*A3* and S1*A*). The equivalent values for shUBF72 were 0.33 ± 0.06 and 0.21 ± 0.02 (Fig. 1, *A2* and *A3*), respectively. Both UBF1 and UBF2 were downregulated as the shRNA construct targets both alternatively spliced isoforms (see further validation controls in Fig. S1, *B1* and *B2*). Quantification of total *Ubf* mRNA expression in the two shUBF clones showed consistently significant reductions relative to WT, specifically 0.55 ± 0.05 for shUBF16 (Fig. 1*B1*) and 0.40 ± 0.04 for shUBF72 (Fig. 1*B2*). Thus, both shUBF16 and shUBF72 exhibited UBF levels that could be compared with a haploinsufficiency status, known to be viable in mice (6); given the essential role of UBF in rRNA transcription, further reduction beyond some critical level would likely no longer be viable.

**Figure 2:**
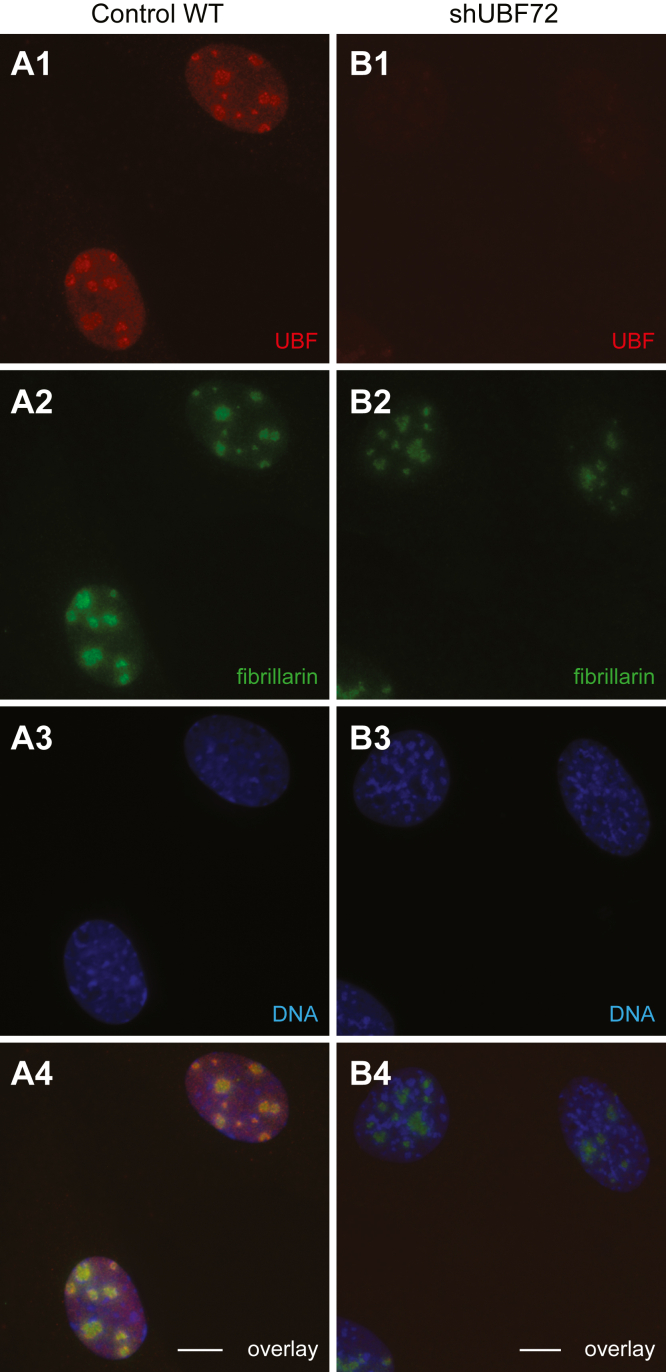
**Immunofluorescence characterization of shUBF72**. Representative images of UBF (*red*) and fibrillarin (*green*) immunodetection in the nucleolus, comparing WT (*panels A1–A4*) and shUBF72 (*panels B1–B4*). DNA is counterstained with Hoechst (*blue*) and an overlay of all fluorophores is displayed in the bottom row. Immunolabeling was carried out in parallel and image acquisition parameters were kept constant in all cases to allow direct comparison of immunofluorescence signals between cell lines. A pronounced reduction of the UBF signal is visible in shUBF72 (*B1*) while the overall architecture of the nucleolus is similar to WT. Scale bars 10 μm.

Furthermore, UBF immunofluorescence signals in the nucleolus of both shUBF16 and shUBF72 cells were markedly reduced compared with WT cells (Fig. 2; compare panel *B1* with *A1*; not shown for shUBF16) while nucleolar marker fibrillarin appeared only slightly reduced (Fig. 2 *A2*–*B2*) and the overall nucleolar organization seemed intact (Fig. 2, *A4*–*B4* in overlays). Consistently, quantification of P53 protein levels, phosphorylation (Ser392), and gene expression did not indicate that UBF downregulation in shUBF72 was associated with p53-dependent nucleolar stress; in particular, both at base line conditions and with experimentally induced nucleolar stress by treatment with actinomycin, P53, pP53 protein levels, and *P53* mRNA were considerably reduced in shUBF72 relative to WT cells (Fig. S1, *C1–C4*). Additionally, gene expression of P53 downstream target protein P21 in shUBF72 was also moderately reduced compared with WT (Fig. S1*C4*). Quantification of cell proliferation revealed significantly lower growth rates for shUBF72 cells compared with WT (Fig. S1*D*). Cell cycle analysis indicated an altered profile with a clear delay in the G1 phase for shUBF72 and a consequent increase in the fraction of the cell population in G1 by a near 40% and corresponding decreases in phases S (45%) and G2/M (59%), compared with WT (Fig. S1, *E1* and *E2*). Delay of cells in G1 is typically associated with impairment in ribosome biogenesis (38).

**Figure 3:**
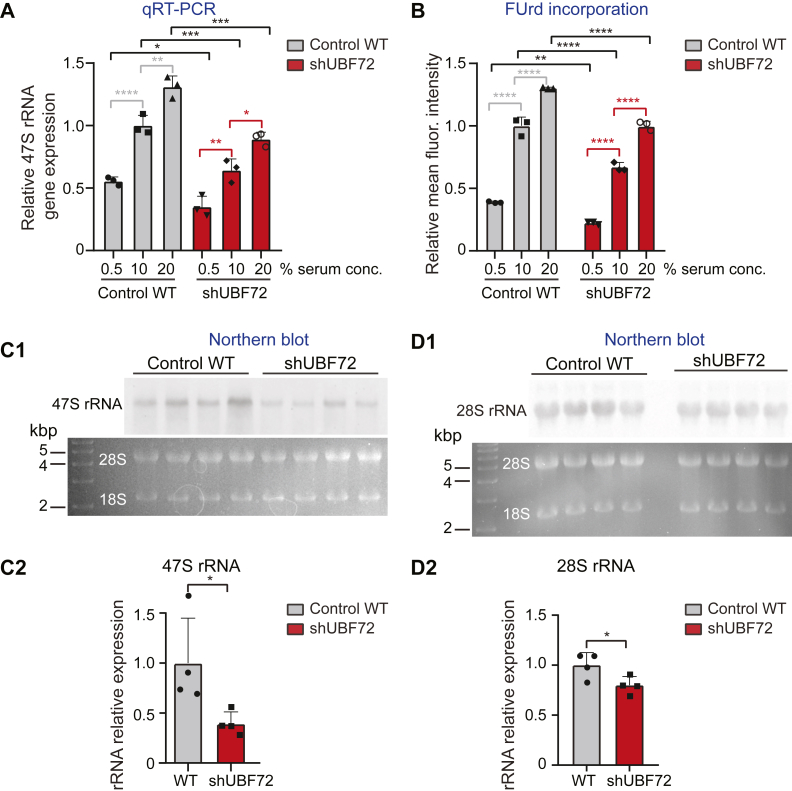
**Quantification of rRNA in shUBF72 shows reduced expression.***A*, quantification of relative pre-rRNA precursor 47S rRNA expression by qRT-PCR comparing WT and shUBF72 cells grown in parallel under three different serum concentrations in culture medium (0.5%, 10%, and 20%), each from three independent experiments. Results show that both in WT and in shUBF72, production of 47S rRNA is serum dependent and its expression is reduced in the shUBF72 clone at all serum concentrations, compared with WT values. The statistical significance of differences was assessed by two-way ANOVA with Tukey’s correction. *B*, quantification of FUrd incorporation into newly transcribed RNA in the nucleolus, in the same experimental setup, mirrors the results in (*A*), displaying serum dependency of incorporation and decreased incorporation in shUBF72 at all serum concentrations, relative to WT. Statistical significance of differences was assessed by two-way ANOVA with Tukey’s correction. *C1* and *C2*, Northern blot analysis of 47S rRNA levels (*panel C1*; 20 μg total RNA loaded per lane) and its quantification (*panel C2*). In *C1*, hybridization of a set of 4 independent experiments with a 47S rRNA-specific probe shows a visible reduction in signal (*upper panel*) with corresponding ethidium bromide staining of the gel, to display major rRNA species 28S and 18S (*lower panel*). Quantification confirms a significant reduction of 47S rRNA in shUBF72. *D1* and *D2*, corresponding analysis for 28S rRNA in the same samples as in *C1* (*panel D1*; 3 μg total RNA loaded per lane), reveals a significant reduction of 28S rRNA in shUBF72 (*panel D2*). The statistical significance of differences in *C2* and *D2* was assessed by Student’s *t* test.

Given the significant reduction of UBF expression in the shUBF cell lines and the critical role of UBF in orchestrating the transcription of the rRNA genes, a marked reduction in the production of rRNA would be expected in these cells and we assessed this by different approaches. First, a quantitative RT-PCR analysis was conducted in 3 independent experiments, using as primers an oligonucleotide pair targeted to the extreme 5′ of the 47S rRNA precursor that can specifically detect the primary rRNA transcript (Table S1). This analysis was extended to include samples taken at 3 different growth conditions, with WT and shUBF72 cells exposed to the restricted growth medium (0.5% serum concentration), normal medium (10% serum), and stimulated growth medium (20% serum). We observed that relative levels of 47S rRNA expression in shUBF72 were clearly reduced as compared with WT values at all serum concentrations (Fig. 3*A*). Despite this, 47S rRNA expression in shUBF72 increased in step with serum as in WT, suggesting that the mechanisms of growth regulation still functioned normally even after UBF depletion. A similar reduction of pre-rRNA expression at all serum concentrations and dependency of UBF expression on serum concentration was also observed with qRT-PCR analysis of shUBF16 cells (data not shown).

We next employed short pulse labeling of nascent RNA with FUrd to monitor rRNA expression. Again, the experiments were conducted under the three different growth conditions (0.5%, 10%, and 20% serum), and the mean fluorescence intensity in the nucleoli of a large number of cells (n = 50 per condition in each cell line) were compared. Again, significant reductions of FUrd incorporation in shUBF72 (indicative of reduced nascent rRNA production) were observed at all serum concentrations as compared with WT, and dependency of rRNA transcription on serum concentration was registered in both WT and shUBF cells (Fig. 3*B*).

In complement, Northern blot analysis, which allowed the resolution of distinct rRNA species, confirmed, in four independent experiments, the reduction of 47S rRNA in shUBF72 (Fig. 3, *C1* and *C2*) as well as of 28S rRNA, the largest mature rRNA species resulting from the multi-step processing of the 47S rRNA precursor (Fig. 3, *D1* and *D2*).

Overall, therefore, the UBF-depleted clones, shUBF16 and shUBF72, displayed robust reduction of *Ubf* mRNA and protein levels and a consequent decrease in rRNA production and were thus reliable tools for analysis of further aspects of rRNA transcription regulation.

### Repressed growth signaling with a reduction of key effectors is linked to the downregulation of UBF

Pol1-driven transcription of rRNA is subject to an intricate network of regulation in response to extracellular stimuli (signaling cues) or intracellular factors (cell cycle, cell growth) (see introductory comments). These include different signaling cascades that target factors of the transcription machinery; for example, the major growth- and proliferation-regulating PI3K/mTOR and RAF-MEK-ERK (MAPK) pathways both modulate the phosphorylation status of UBF, among others, thus affecting rRNA transcription initiation, elongation, and chromatin configuration. We thus set up assays to quantify mTOR and MAPK signaling under UBF downregulation in a manner that would preserve native protein phosphorylation and ensure specificity (with the use of inhibitors rapamycin for mTOR C1 kinase and U0126 for MAPK), comparing WT and shUBF72 cells in independent experiments, in parallel.

**Figure 4:**
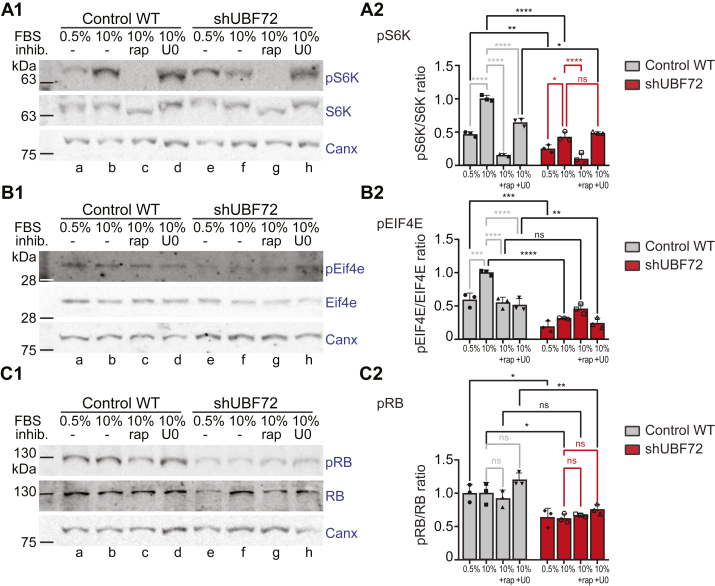
**Growth signaling and cell cycle factors are reduced in UBF silencing conditions.***A1* and *A2*, representative WB analysis (*panel A1*) and quantification of three independent experiments (*panel A2*) for pS6 kinase, expressed as a fraction of total S6K (pS6K/S6K), comparing WT and shUBF72 and using mTOR inhibitor rapamycin (rap) and MAPK inhibitor U0126 (U0). In the WB, the detection of pS6K, total S6K, and calnexin (used as loading control for normalization of all samples) is displayed. The quantification shown in *A2* depicts the average normalized values of pS6K/S6K protein and reveals a significant decrease in the fraction of pS6K protein compared with WT cells (at 0.5 and 10% serum). Upon rapamycin treatment, pS6K/S6K is reduced in both shUBF72 and WT cells, compared with 10% serum conditions. Upon U0126 treatment, pS6K/S6K is reduced in WT cells while slightly increased in shUBF72. Shown are the average values ± SD of three independent experiments. *B1* and *B2*, equivalent experiment as above, analyzing the pEIF4E/EIF4E fraction, shows a significant decline in the ratio of shUBF72 at both 0.5 and 10% serum. *C1* and *C2*, equivalent analysis for cell cycle regulator RB, expressed as a pRB/RB fraction. Although pairwise differences (reduction in shUBF72) are not always statistically significant, a reduction of pRB/RB in shUBF72 at 0.5% and 10% serum and also in the presence of U0126, relative to WT, is clear. The statistical significance of differences in A2-C2 was assessed by two-way ANOVA with Tukey’s correction.

S6 kinase (S6K) is a prominent direct downstream target of mTOR kinase; under growth stimulation, activated mTOR kinase phosphorylates S6K which, in turn, phosphorylates UBF, thus boosting rRNA transcription (39). Consistently, when we quantified the fraction of pS6K relative to total S6K (pS6K/S6K) in WT cells, we observed a doubling of pS6K/S6K from low to high serum concentration (Fig. 4*A1* for representative WB and *A2* for quantification; compare the ratio at 0.5% and 10% serum), a near elimination in the presence of rapamycin, and substantial reduction in the presence of U0126 (Fig. 4, *A1* and *A2*). Serum and mTOR dependency were replicated in shUBF72 but, interestingly, at all serum concentrations and conditions, the pS6K/S6K ratio was markedly reduced (>50% of WT at 10% serum; Fig. 4*A2*). This suggested downregulation of the growth signaling axis and specifically of the mTOR kinase pathway under UBF depletion.

Quantification of another marker of mTOR activation, pEIF4E/EIF4E, gave similar results. Elongation initiation factor EIF4E is activated by MAPK-dependent phosphorylation and mTOR-dependent phosphorylation and release of its repressor factor 4E-BP1 (40, 41). Again, in WT cells the pEIF4E/EIF4E ratio showed serum dependency and was decreased in the presence of either rapamycin or U0126 (Fig. 4, *B1* and *B2*). In agreement with reduced growth signaling in shUBF72, the pEIF4E/EIF4E ratio was markedly lower in UBF-depleted cells at all conditions, relative to WT, and in particular at 10% serum it was, again, 50% of WT (Fig. 4*B2*). These observations would point to the downregulation of growth signaling as well as translational competence on UBF depletion.

We next quantified a known tumor suppressor and cell cycle regulator, Retinoblastoma (RB), also known to impact rRNA transcription; non-phosphorylated RB sequesters UBF, while pRB releases it, thus boosting rRNA production (42, 43, 44). Interestingly, pRB/RB ratios were significantly reduced in shUBF72 relative to WT but in neither WT nor shUBF72 did we observe notable fluctuations in the ratios across the different conditions (Fig. 4, *C1* and *C2*). Downregulation of pRB was thus another intracellular cue linked to repressed rRNA transcription in shUBF.

Together, these experiments are evidence of significantly repressed growth signaling accompanied by loss of transcriptional and translational efficiency in cells when UBF is reduced. Since this signaling is upstream of UBF, our findings suggest a bidirectional feedback between both extrinsic and intrinsic growth signaling and rRNA biosynthetic control.

### Reduction of the fractions of both phosphorylated UBF1 and UBF2 and decreased chromatin fractional occupancy accompanies UBF paucity

**Figure 5:**
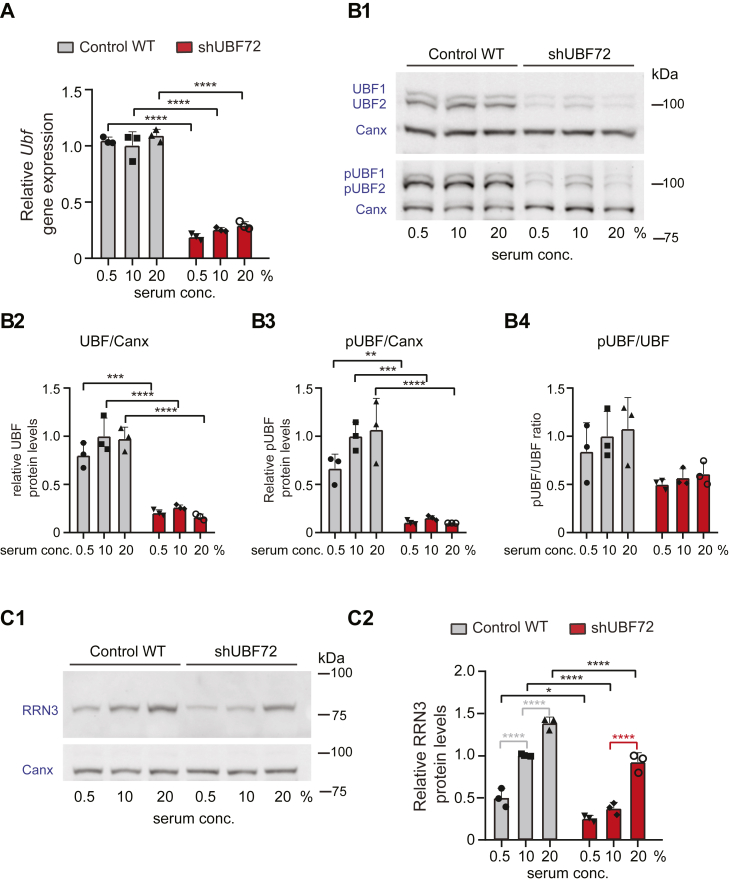
**Quantification of *Ubf* gene expression and protein levels, levels of the pUBF fraction and RRN3 in shUBF72, and their serum dependency.***A*, quantification in three independent experiments of relative expression of *Ubf* by qRT-PCR comparing WT and shUBF72 cells grown in parallel under three different serum concentrations in culture medium (0.5, 10, 20%), shows insignificant fluctuations within each cell type, albeit with great and statistically significant reduction in shUBF72, relative to WT, at all serum concentrations. *B1*, corresponding WB analysis from three independent experiments of protein levels of total UBF confirms this finding (*upper panel*). The same samples were re-run on a new blot and pUBF signal was detected with a phospho-S484-specific antibody (*bottom panel*). *B2–B4*, quantification of results from these 3 independent experiments with normalization of the total UBF signal (UBF1+2) to same-lane calnexin (*B2*), normalization of the total pUBF signal (pUBF1+2) to same-lane calnexin (*B3*), and calculation total pUBF signal as a fraction of total UBF (pUBF/UBF) (*B4*). *C1* and *C2*, representative WB analysis (*C1*) and quantification (*C2*) of protein levels of RRN3 factor in three independent experiments, under the three different serum concentrations and normalization to same-lane calnexin, shows serum-dependency and a significant reduction in shUBF72, relative to WT samples. The statistical significance of differences was assessed by two-way ANOVA with Tukey’s correction in all panels.

The next part of our analysis focused on UBF itself. We first investigated what happens to UBF levels when cells are challenged with different growth conditions; at 0.5% serum starvation, standard 10% serum, or stimulation with 20% serum. Quantitative RT-PCR in 3 independent experiments revealed that *Ubf* gene expression remained essentially unaltered under all growth conditions for both WT and shUBF cells, despite *Ubf* mRNA being severely downregulated to levels well below 50% in shUBF72 (Fig. 5*A* and, as shown for 10% serum, in Fig. 1*B2*). The same was the case for UBF protein levels (for both UBF1 and UBF2), as observed when the average measurements at each condition were normalized to same-sample calnexin protein levels (Fig. 5, *B1* and *B2*), suggesting only minor fluctuations in absolute protein concentrations. We also observed stable gene expression of UBF and a constant ratio between UBF1 and UBF2 isoforms throughout the cell cycle phases in synchronized WT cells, indicating no differential pre-mRNA splicing (Fig. S2).

The finding of stable UBF expression may not be surprising given that it is well established that UBF-mediated PIC formation is, rather, dependent on UBF phosphorylation (23). We thus next quantified the fraction of UBF phosphorylated at Ser 484 (pUBF) in 3 independent experiments with cells grown under the three serum conditions. Similar to total UBF1 and UBF2, the protein levels of pUBF1 and pUBF2 were significantly reduced in shUBF72 at all conditions, relative to WT (Fig. 5*B3*). A trend for serum-responsive pUBF increase within WT or shUBF72 cells, also when pUBF was shown as a fraction of total UBF protein (pUBF/UBF) (Fig. 5*B4*), was not statistically significant and it is not clear whether it is biologically relevant. These results indicated that limited absolute levels of UBF in cells were accompanied by concomitant diminution of the transcription-enhancing pUBF levels. Given that pUBF functions synergistically with factor RRN3 to assemble the PIC and initiate rRNA transcription, we also assessed RRN3 protein levels under the same experimental set up (Fig. 5*C1*). We observed RRN3 concentration to display serum-dependent modulation and a severe decrease in shUBF72 under all serum concentrations, relative to WT cells (Fig. 5*C2*).

**Figure 6:**
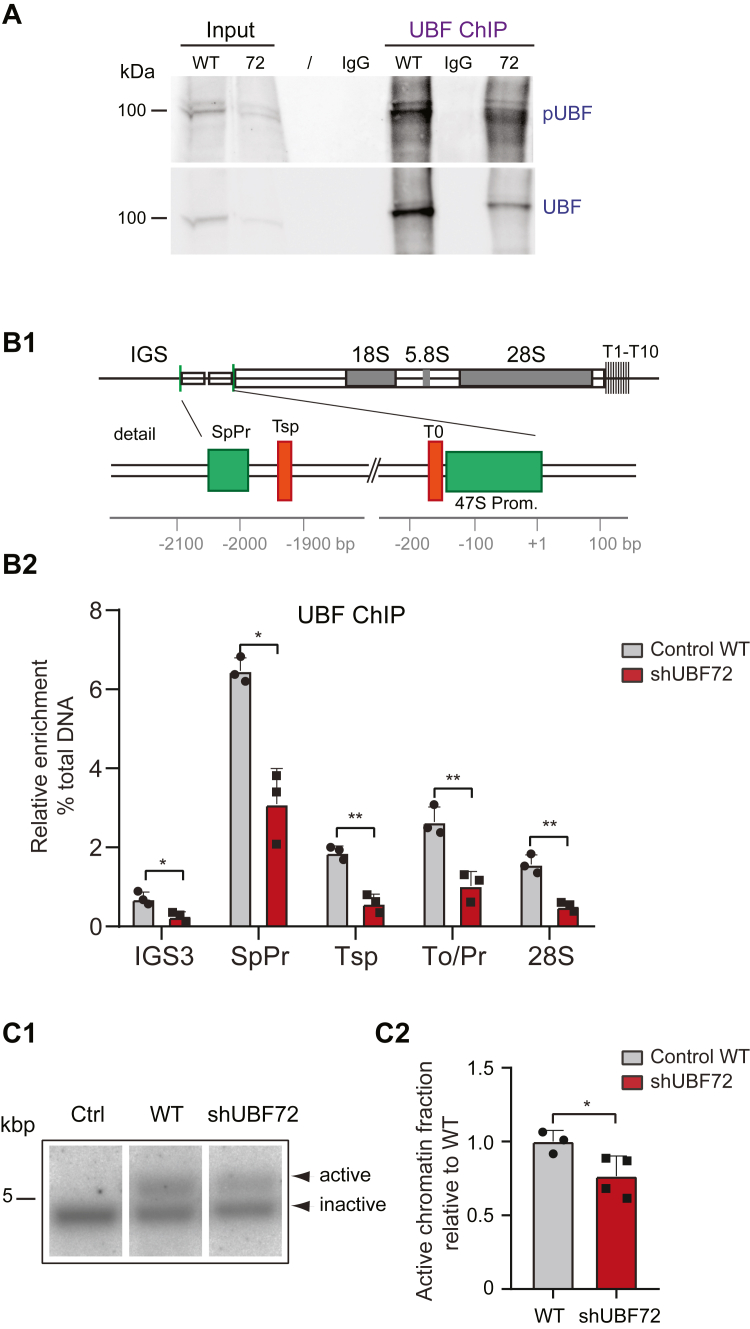
**Analysis and quantification of UBF binding to rDNA chromatin.***A*, WB analysis of fractions used in the UBF ChIP experiments shown in *B2*. Representative WB: “Input” corresponds to 20% of the total input samples derived from cross-linked cell lysates; “UBF ChIP” samples correspond to an equal amount of the amount used for ChIP in *B2*. The IgG sample corresponds to negative control and the (/) symbol denotes no sample loading in that lane. Strips were probed with anti-pUBF (*upper panel*) or anti-UBF antibodies (*lower panel*). *B1*, schematic representation of the functional 45kb mouse rDNA repeat unit (*upper panel*) to illustrate the constituent parts, and detail of the enhancer/promoter area (*lower panel*), to indicate the DNA sequence elements to which oligonucleotide primers were targeted for the qPCR in the UBF ChIP quantification shown in *B2* (IGS3, intergenic spacer 3; SpPr, spacer promoter; Tsp, terminator spacer; To/Pr, To promoter proximal terminator/promoter; 28S, 28S rRNA coding sequences; T1-T10, terminator sites 1–10). *B2*, UBF ChIP quantification of 3 independent experiments to compare UBF occupancy in shUBF72 and WT at known UBF binding sites across the rDNA unit shows reduced occupancy in shUBF72 at all sites. For example, for the SpPr hotspot UBF binding is 3.09 ± 0.91 for shUBF72 vs. 6.48 ± 0.33 SD (or 47% of WT). The statistical significance of differences was assessed by multiple *t* tests using the Holm-Sidak correction with α = 0.05. *C1* and *C2*, representative example of Southern blot analysis of “open`’ (or active/potentially active, transcriptionally competent) rDNA chromatin segment (*upper band*) and “closed” (or inactive/silent) rDNA chromatin segment, following psoralen incorporation and crosslinking experiments, comparing WT and shUBF samples (*C1*; Ctrl, control reference of non-crosslinked DNA). Quantification of four independent experiments shows a statistically significant decrease in the fraction of open chromatin, by 23.41% relative to WT, in shUBF72 (*C2*). The statistical significance of differences was assessed by Welch’s *t* test.

We next employed chromatin immunoprecipitation experiments (ChIP), which specifically allow the assessment of the chromatin-bound UBF fraction, using total input samples (“input”) from cross-linked cells, and eluted samples, following UBF immunoprecipitation for qPCR (“ChIP” samples) (shown by WB analysis in Fig. 6*A* to monitor UBF and pUBF). UBF occupancy of rDNA chromatin, at binding sites over the whole rDNA unit as now quantified by qPCR (Fig. 6*B1*), was clearly reduced in shUBF72 compared with WT at all positions, including regulatory and coding regions (Fig. 6*B2*). In particular, known UBF-binding hotspots in the promoter-enhancer sequences and also binding within the 28S rRNA coding region displayed UBF occupancy that was reduced to around 40% in shUBF72 relative to WT (Fig. 6*B2*).

UBF is responsible for the non-nucleosomal conformation of rDNA chromatin and this “active” or “poised” state of chromatin, which can be considered transcriptionally competent, can be directly monitored by psoralen accessibility crosslinking (PAC) (2, 3, 6). The PAC assay revealed that depletion of UBF in shUBF72 cells induced an almost 25% reduction in the fraction of potentially active rDNA copies (Fig. 6, *C1* and *C2*). The reduction of the gene fraction with permissive chromatin status combined with the reduction of UBF occupancy of rDNA chromatin detected with ChIP assays was consistent with, and explained, the decline in active 47S rRNA synthesis observed in these cells (Fig. 3, *A* and *B*).

### RNA pol1 cellular concentration is modulated as a target of rRNA transcriptional control mechanisms

Effects on promoter binding by RNA polymerases generally, including RNA Pol1, and the modulation therefore of transcription initiation rate, is a typical and well-characterized mode of transcriptional regulation. Here, we investigated what happens to the pool of available cellular Pol1 under conditions of UBF depletion. We quantified separately two distinct Pol1 subunits: PolR1E (RPA49/PAF53), a 53kDa-protein of the TFIIE/F-like heterodimer component of Pol1 that mediates its interaction with UBF, and PolR1A (RPA190) the 194 kDa largest Pol1 subunit at its catalytic core (14, 25).

**Figure 7:**
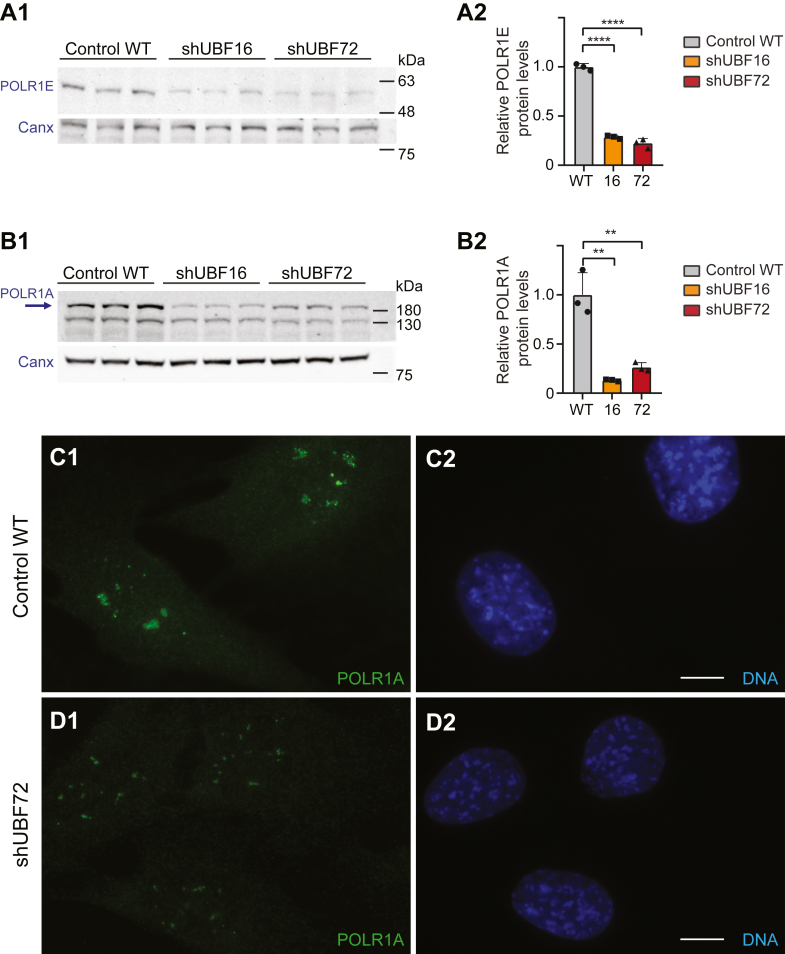
**Protein levels of RNA Pol1 are reduced upon UBF silencing.***A1* and *A2*, analysis of protein levels of RNA Pol1 subunit POLR1E by WB shows a profound decrease in both shUBF16 and shUBF72, relative to WT cells. WB of three independent experiments is displayed in *panel A1* and the corresponding quantification in *panel A2* with protein levels normalized to calnexin. See Fig. S3*panels A1+A2* for alternative normalization to total protein, as visualized by Ponceau staining, and Fig. S3*panels B1–B3* for WB and quantification of actin, as a negative control (no change) from the same experimental set. *B1* and *B2*, WB (*panel B1*) and corresponding quantification (*panel B2*) of RNA Pol1 subunit POLR1A in 3 independent experiments. Relative POLR1A protein levels are normalized to calnexin, and the decrease is large in both shUBF cell lines. See Fig. S3*panels C1+C2* for alternative POLR1A quantification to Ponceau staining from the same experimental set. The statistical significance of differences in *A2* and *B2* was assessed by one-way ANOVA with Dunnett’s method. *C1–D2*, immunofluorescence detection of POLR1A (*green*) confirms a clear reduction of signal in shUBF72, compared with WT cells (compare *panels C1* and *D1*). Cells were counterstained for DNA (*blue; panels C2* and *D2*), and image acquisition parameters were identical per fluorochrome, across samples. Scale bars 10 μm.

Interestingly, in three independent experiments, we found protein levels of both PolR1E (Fig. 7, *A1* and *A2*) and PolR1A (Fig. 7, *B1* and *B2*) reduced by UBF depletion, in both shUBF16 and shUBF72, to well below 50% of WT levels. This was the case when Pol1 subunit protein normalization was carried out to either calnexin (Fig. 7, *B2* and *C2*) or to total cellular protein as revealed by Ponceau staining (Fig. S3, *A1*, *A2*, *C1*, and *C2*) (and see negative control experiment with unchanged actin protein levels in the same samples, in Fig. S3, *B1* and *B2*). In further support, the immunofluorescence signal for PolR1A localization in the nucleolus in shUBF72 cells was noticeably weaker and more restricted, compared with WT cells (Fig. 7, compare panels *C1* and *D1*).

**Figure 8:**
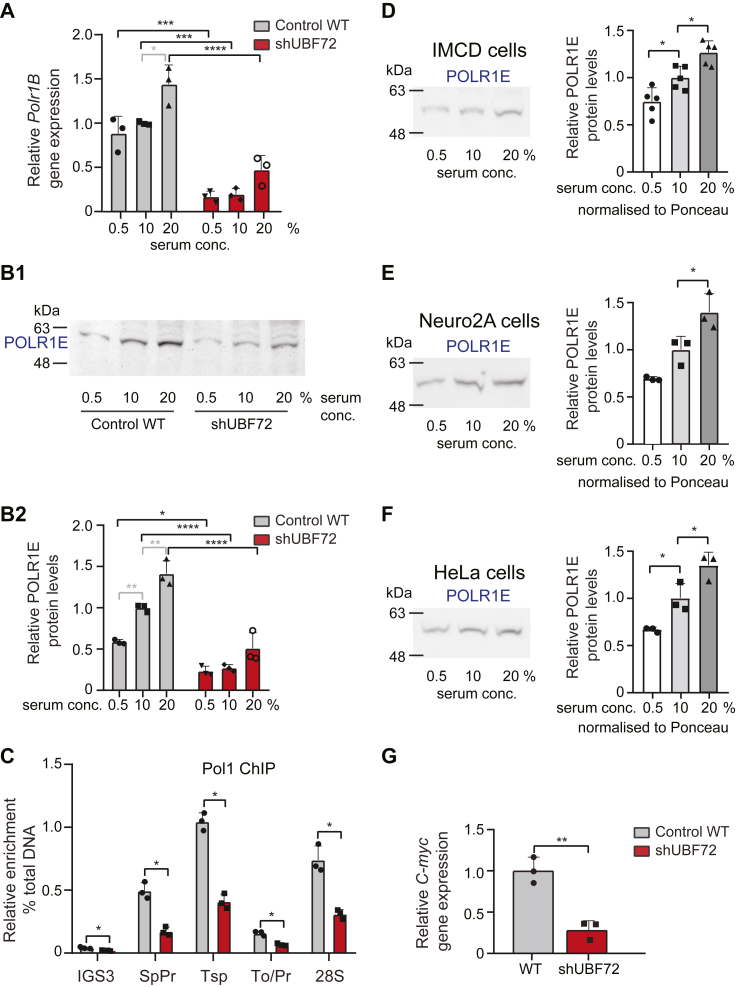
**RNA pol1 is modulated at the transcriptional level by extracellular growth signals in different WT cells and its expression is markedly affected by UBF silencing.***A*, quantification in three independent experiments of relative expression of *Polr1B* subunit of RNA Pol1 by qRT-PCR comparing WT and shUBF72 cells grown in parallel under three different serum concentrations in culture medium (0.5%, 10%, and 20%), reveals a serum-dependent increase in WT and a similar tendency and a clear corresponding reduction of *Polr1E* mRNA levels in shUBF72 at all serum concentrations. Statistical significance of differences was assessed by two-way ANOVA with Tukey’s correction. *B1* and *B2*, representative WB (*panel B1*) and quantification of POLR1E protein levels (*panel B2*) in three independent experiments of an equivalent setup confirms the serum-dependent modulation of RNA Pol1 in both WT and shUBF72. Sample normalization was to same-sample total protein as revealed by Ponceau whole-membrane staining (see Ponceau stained gel in Fig. S4*A*). Statistical significance of differences was assessed by two-way ANOVA with Tukey’s correction. *C*, RNA Pol1 ChIP quantification of 3 independent experiments to compare Pol1 occupancy in shUBF72 and WT at known binding sites across the rDNA unit (with oligonucleotide primers shown in Fig. 6*C1*) shows reduced occupancy in shUBF72 at all sites to a range of 40% of WT or lower. Statistical significance of differences was assessed by multiple t-tests using the Holm-Sidak correction with α = 0.05. *D*, representative WB of POLR1E levels in WT mouse IMCD cells, grown at different serum concentrations, and quantification of 5 independent experiments with sample normalization to its Ponceau staining (see Ponceau-stained gel in Fig. S4*B*). *E*, equivalent experiment using mouse Neuro2A cells, with corresponding Ponceau gel in Fig. S4*C*. *F*, equivalent experiment using human HeLa cells, with corresponding Ponceau gel in Fig. S4*D*. Statistical significance of differences in (*D*–*F*) was assessed by one-way ANOVA with Dunnett’s method. *G*, quantification of relative gene expression of the *C-myc* oncogene in WT and shUBF72 cells in 3 independent experiments, shows a significant decrease of *C-myc* mRNA levels upon UBF silencing. Statistical significance of differences was assessed by Welch’s *t* test.

Because modulation of levels of the polymerase itself is a rather atypical manner of transcriptional regulation, we were interested in further characterizing this finding and we therefore next examined whether reduced protein levels could be attributed to enhanced protein instability (due to the downregulation of protein interactor UBF) or more upstream transcriptional control of Pol1 gene expression. Quantification of gene expression of yet another Pol1 core subunit, PolR1B (RPA135), conducted under the three serum regimes, clearly showed very strong downregulation in shUBF72 at all conditions. For instance, at 10% serum, relative *Polr1B* mRNA levels in shUBF72 were reduced 5.3-fold (0.19 ± 0.07 vs. 1.0 ± 0.02 in WT), to 19% of WT levels. (Fig. 8*A*). Additionally, an upward trend in *Polr1B* mRNA accompanied serum elevation, in both WT and shUBF72 cells (Fig. 8*A*). These results were mirrored by equivalent experiments, quantifying the corresponding changes and serum dependency of POLR1E at the protein level (Figs. 8, *B1* and *B2* and S4*A*). For example, at 10% serum, POLR1E protein levels were reduced to 18% of WT (0.18 ± 0.02 in shUBF72 vs. 1 ± 0.2 in WT). These combined findings, therefore, indicated that the cellular concentration of Pol1 was regulated at the transcriptional level by growth conditions/signaling as well as by the depletion of UBF.

Consistent with reduced rRNA transcriptional output and reduced Pol1 protein levels, ChIP experiments using anti-pol1 antibody to POLR1A revealed reduced fractional occupancy for chromatin-associated Pol1 across the rDNA unit (Fig. 8*C*). In particular, Pol1 occupancy over the Sp/Pr and T0/Pr promoter sequences and the 28S rRNA coding sequences were, respectively, 0.18 ± 0.03 in shUBF vs. 0.50 ±0.07 in WT (or 36% of WT), 0.41 ±0.06 vs. 1.05 ± 0.07 (or 39% of WT), and 0.07 ± 0.01 vs. 0.16 ± 0.02 (or 44% of WT) (Fig. 8*C*). The reduced Pol1 occupancy in shUBF72 was consistent with the observed relative 47S rRNA synthesis in these cells.

The modulation of RNA Pol1 concentration in shUBF cells was rather unexpected and hence we wished to investigate whether this was a more generalized mechanism of rRNA transcriptional control in proliferating cells. We therefore assessed POLR1E protein levels at increasing serum concentrations in mouse or human cell lines other than NIH 3T3 fibroblasts, namely the mouse IMCD kidney cell line (Fig. 8*D*) and Neuro2 neural crest-derived cell line (Fig. 8*E*), as well as the human HeLa cell line (Fig. 8*F*). Interestingly in all these wild-type cell lines of different cell types and species, we confirmed significant changes in the protein levels of POLR1E (normalized to total protein as per Ponceau, Fig. S4, *B*–*D*), in step with increased serum concentration (Fig. 8, *D*–*F*).

**Figure 9:**
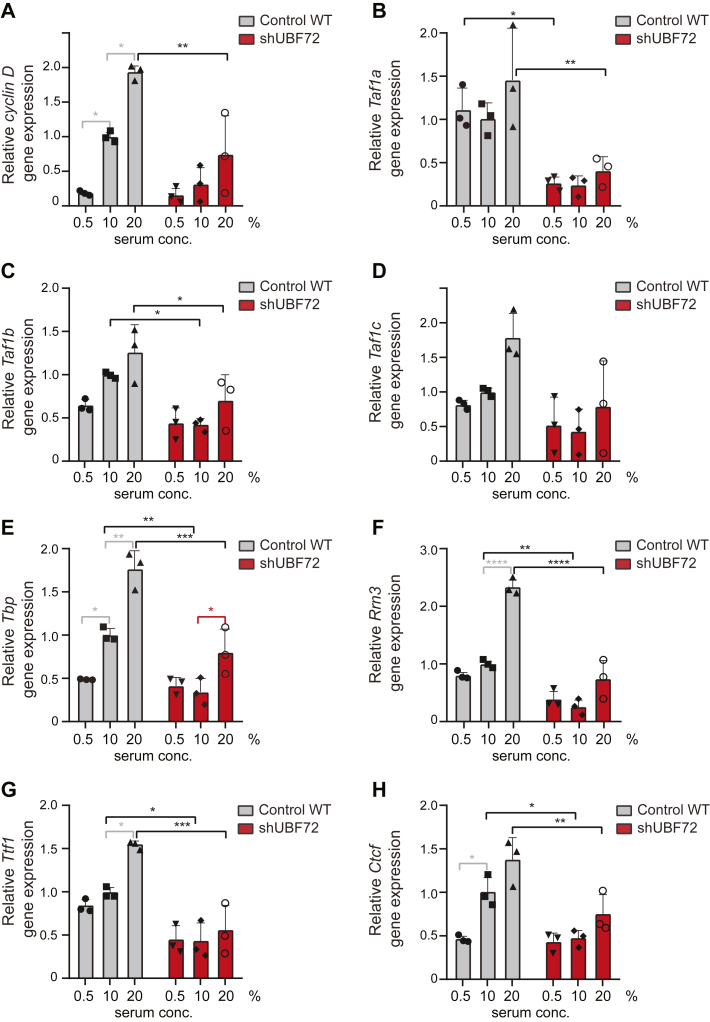
**Assessment byqRT-PCR of how UBF silencing affects gene expression of factors that are critical for rRNA transcription**. *A*, quantification in three independent experiments of mRNA levels of *Cyclin D* in WT control and shUBF72 cells grown in parallel at three different serum concentrations in the culture medium (0.5%, 10%, and 20%). *B–E*, quantification in the same samples of mRNA levels of components of the PIC: *Taf1a* (*panel B*), *Taf1b* (*panel C*), *Taf1c* (*panel D*) and *Tbp* (*panel E*). *F*, quantification in the same samples of mRNA levels of PIC-interacting factor *Rrn3*. *G* and *H*, quantification in the same samples of mRNA levels of termination factor *Ttf1* (*panel G*) and upstream element-binding and chromatin remodeler factor *Ctcf* (*panel H*). To be directly comparable, values were calculated using the common base method (71) and expressed as a ratio to the normalized mean WT value at 10%, with error bars corresponding to SD. The statistical significance of differences in all panels was assessed by two-way ANOVA with Tukey’s correction.

To gain some initial understanding of the molecular mechanisms involved in the regulation of Pol1 gene expression, we interrogated a potential role for the C-MYC transcription factor. C-MYC has been shown to co-ordinate gene expression of Pol1 subunit POLR1B, PIC factor RRN3, and UBF itself, through binding to their respective promoters, and has also been found to affect rRNA transcription *via* direct interaction with rDNA regulatory elements (45, 46, 47, 48). We found that gene expression of *C-myc* was drastically reduced in shUBF72 compared with WT cells (Fig. 8*G*), correlating its downregulation with the observed reductions in Pol1 subunits. Given the link between *C-myc* expression and a larger number of genes associated with Pol1 transcription by transcriptome analysis in stages of granulocyte differentiation (46), we further quantified the expression of eight highly relevant genes in WT and shUBF72 cells, grown at increasing serum concentrations (Fig. 9, *A*–*H*). Specifically, we monitored the expression of Cyclin D, important for UBF S484 phosphorylation by CDK4 and re-activation of rRNA transcription after mitosis (18), of SL1-subunits TAF1A, 1B, 1C, the TBP, and RRN3 (TIF1A; whose protein levels we found to be downregulated upon UBF depletion in Fig. 5, *C1* and *C2*), as well as transcriptional terminator/chromatin modifier TTF1 and CTCF, a non-PIC modifier of rDNA structure. In all instances, quantification showed a clear trend of increased gene expression of each of these factors, as a function of serum concentration in both WT and shUBF72 cells, and all showed a large decrease of expression in shUBF72, relative to WT, at each serum concentration (Fig. 9, *A*–*H*).

Interestingly, as we have shown here, the functional interactions affected by UBF depletion include factors that are both upstream, at the same level, and downstream of UBF. Additional confirmation of this network of functional interactions of UBF was provided by the ability of a cocktail of exogenous UBF1 and UBF2 to rescue downstream and upstream UBF depletion effects. Specifically, transient transfection of shUBF72 cells with a plasmid mix expressing shUBF72 oligo-resistant, synonymous triple mutant FLAG-tagged UBF1 and UBF2 (Fig. S5, *A1*–*A4*) fully restored the protein levels of POLR1E subunit of RNA Pol1 (Fig. S5, *B2* and *B3*) and substantially rescued expression of *C-myc*, compared with shUBF72 cells transfected in parallel with an empty FLAG vector (Fig. S5*C*). Finally, in a complementary approach, we constructed a third UBF-silenced cell line, named shUBF555, now targeting a different site of the UBF1/2 transcripts than shUBF72 and shUBF16 (close to the 5′ end as opposed to the 3′ end by shUBF72/16, Fig. S6*A*) and displaying similar levels of UBF depletion (Fig. S6, *B1* and *B3*). shUBF555 recapitulated the phenotypes we documented for shUBF72 and shUBF16. In particular, shUBF555 exhibited significantly reduced mRNA and protein levels of RNA pol1 (Fig. S6, *C1* and *C3*), markedly reduced pre-rRNA levels (Fig. S6*D1*), as well as downregulated gene expression of *Rrn3* (Fig. S6*D2*) and of upstream transcription factor *C-myc* (Fig. S6*D3*).

In the overall conclusion of our work, the coordinate downregulation of these proteins suggests that downregulation of UBF alone leads to a drastic and large-scale re-adaptation of their expression and reveals an unrecognized network coordinating the expression of the critical factors that control rRNA expression.

## Discussion

The multi-layered involvement of UBF in rRNA transcription has long been the subject of intense effort and discovery. Originally identified as the first general factor specific for Pol1, this multi-HMG box factor has been shown to perform distinct roles in transcription promotion, chromatin remodeling, gene regulation, and epigenetic programming (see introductory comments). More recently, heterozygous *de novo* gain-of-function missense mutations in human *UBF* (E210K) that upregulate rRNA transcription have been identified as the cause of severe developmental neuroregression in childhood ((49, 50); also the Q203R mutation in (51)). Introduction of homozygous E210K mutations in mouse knock-in MEFs, in contrast, reduced rDNA transcription rates (3). In addition, *UBF* tandem insertions and gene fusions have been linked to acute myeloid and lymphoblastic leukemias (52, 53). These studies thus broadened the scope of interest in UBF, its molecular and cellular functions, and the impact of rRNA deregulation in disease mechanisms.

In this work, first, we provide evidence that UBF depletion results in a significant reduction of rRNA transcription, even when UBF is in a quasi-haploinsufficiency status in cells. The effect of UBF depletion on rRNA synthesis has been a point of discussion in the past, and our work is in line with evidence documenting the link between UBF downregulation and rRNA transcriptional reduction (2, 6). Our UBF partially-depleted cellular model allowed us to take a broader view of the rRNA transcriptional machinery as a whole. Our results indicate that, in the absence of P53-induced nucleolar stress or obvious disruption of nucleolar integrity, UBF partial depletion simultaneously affects all factors known to affect rRNA transcription, both at the initiation stage (the SLI components and RRN3), and also in chromatin organization relating to its permissibility to transcription (TTF1, CTCF) and Pol1 itself. The protein-protein interactions and functional interrelationships between UBF and some of these factors are well known; it is established that UBF is essential for the recruitment of all components of the RPI transcription machinery and these interactions are usually quantified on the basis of factor binding to rDNA chromatin (as a proxy for transcription-competent chromatin configuration). Here, surprisingly, we find reduced chromatin recruitment to result from a reduction in protein levels of these transcription machinery components, as a consequence of UBF paucity. Reduction in protein levels does not appear to be a consequence of increased protein instability due to the loss of their interaction partner UBF. Rather, although we have not directly measured protein stability experimentally, we show that the depletion of the RPI machinery is co-ordinately regulated at the transcriptional level for each of these factors, as we demonstrate that gene expression of these factors is also severely restricted when UBF is silenced. Additionally, we observe that the expression of oncogene *C-myc*, which is considered the most upstream factor of the rRNA transcription regulon (45), is similarly affected by UBF depletion. C-MYC is a master regulator implicated in the transcription of all RNA polymerases, Pol I, II, and III, thus affecting all stages of ribosome biogenesis. In particular, C-MYC facilitates the recruitment of SL1 to Pol I promoters and controls the expression of UBF, which is essential for Pol I transcription. It has previously been suggested that C-MYC may be able to silence rRNA genes indirectly, through control of UBF, SL1 factors, and even subunits of Pol1 as their promoters are its direct targets (46) but also through its direct binding at the rDNA promoter (47, 48). C-MYC in part mediates transcriptional activation *via* the recruitment to the promoters of H3 and H4 histone acetyltransferases (47, 48). In our work, we have found a reverse feedback correlation where reduced *Ubf* expression may be able to shut down the rRNA transcription machinery *via* control of *C-myc*. Similarly, UBF depletion seems to impact, in a reverse feedback manner, the upstream mTOR and other growth signaling, whose direct and indirect phosphorylation targets include UBF itself.

How UBF achieves such an extensive transcriptional effect over the entire network, including upstream factors is not clear. However, although UBF has been mostly studied for its critical role in rDNA transcription, both UBF1 and UBF2 are also known to be involved in Pol II transcription. Specifically, they can activate Pol II-mediated transcription and serve as a positive effector in the WNT/β-catenin signaling pathway through cooperation with LEF-1 and β-catenin (35). It is well established that *C-myc* is a target-responsive gene of the WNT/β-catenin pathway (54). Genome-wide analysis in different human cell lines has shown UBF association with a large number of sites in chromatin, with transcription factor and nucleobase metabolism genes, together with genes involved in WNT signaling, being over-represented (55). A further study discovered an additional repertoire of UBTF1/2-bound genes involved in the regulation of cell cycle checkpoints and DNA damage response, including the *C-myc* gene, and demonstrated that UBTF1/2 is required for recruiting Pol II to the highly transcribed histone gene clusters (36). It is therefore conceivable that UBF exerts a much wider range of transcriptional control than previously recognized, including Pol I+II transcription, and in particular, UBF also has a mutually reciprocal effect on the transcriptional regulation of C-MYC.

In this work, we observed that regulation of gene expression levels of RNA Po1 I itself, rather atypically, is another mode of modulating rRNA transcriptional activity. Interestingly, this modulation appears to be serum/signaling-dependent and not restricted to the extreme situation of UBF insufficiency in shUBF cells but also manifested in WT cells which are expressing UBF at normal levels, when stimulated with serum. The observed reduction of Pol1 fractional occupancy in the rRNA promoter sequences of shUBF72 cells is consistent with and possibly a direct result of Pol1 depletion. Pol1 is a highly efficient and very processive enzyme with a rapid initiation and elongation rate calculated to be about 100 nucleotides per second in mammalian cells (56). A classical and well-studied mode of regulation of Pol1 activity is in its rate of promoter binding and transcription initiation; this is typically modulated through the reversible post-translational modifications of PIC factors (*e.g.*, UBF phosphorylation), and it is thought to be rapidly induced and responsive to different stimuli and signaling cascades (reviewed by (57)). The elongation rate of Pol1 can be modulated by its reversible dephosphorylation in yeast and the inclusion of different factor subunits, or the activity of UBF (reviewed by (58, 59)). rDNA is transcribed in long bursts of Pol1 activity (ON-time), punctuated by periods of inactivity (OFF-time), as revealed by real-time imaging; serum starvation or stimulation can modulate burst duration (ON-times) and amplitude (60). Additionally, as we also observe in this work, the rate of rRNA synthesis is modulated *via* changes in the fraction of active rDNA genes from the total pool of rDNA genes, which is achieved through epigenetic modification of the rDNA (61, 62). Such changes are associated with the differentiation status of the cell and this mode of rDNA transcriptional regulation has more long-term effects (reviewed by (63)). In this context, the acute upregulation of RNA Pol1 concentration in response to extrinsic signaling that we report in WT cells, and the more sustained reduction of Pol1 protein levels observed upon UBF restriction in shUBF cells, both regulated transcriptionally, is somewhat surprising. Effects on RNA Pol1 concentration by impacting the protein stability of its subunits through zinc depletion, leading to their degradation by vacuolar proteases, has been reported in yeast (64). However, modulation of RNA Pol1 intracellular concentration at the transcriptional level maybe a less recognized and rarely discussed mode of regulation of rRNA synthesis that may deserve better attention. Indeed, inspection of recent proteomics, transcriptomics and single-cell RNA-seq datasets reveals not only tissue-specific and cell type-specific differential mRNA and protein expression for polymerase subunits POLR1A and POLR1E for instance [W2-W5] but, significantly, that both subunits are also either up- or down-regulated upon different experimental treatments including activation of G-protein-coupled receptors, enzyme-linked receptors, nuclear receptors, protein kinases, transcription factors, or viral infections, [W6-W7]. Thus, availability of RNA Pol1 in the cellular pool, regulated by its own transcription rate, appears to be a *bona fide* additional strategy for short and long-term adaptation of rRNA transcription in cells.

Finally, in this work, we observe that both UBF1 and UBF2 are stably co-expressed throughout the cell cycle in a co-ordinate manner at a stable ratio between them, indicating no differential alternative splicing regulation of the two isoforms (Fig. S2*A1*), both become phosphorylated at Ser486 (Fig. 5*B1*), and both are present across the active rDNA in NIH 3T3 fibroblasts (both WT and shUBF72, Fig. 6*A*; also see (3)). UBF2 is more prominently expressed than UBF1 in NIH 3T3 cells in our hands, both transcriptionally and at protein level. When UBF1 is individually silenced, the protein concentration of UBF2 appears unaffected, suggesting that the protein stability of UBF2 is not negatively impacted by the loss of UBF1 (Fig. S1*B2*, compare lanes a,b, also a’ and b’). *In vivo*, UBF initially was purified from mammalian cells as a complex of UBF1 and UBF2 (65), and both UBF1 and UBF2 were shown to each be capable of forming homodimers but also heterodimers between them, in ratios observed in tissues (31). Additionally, although functional studies assessing the role of UBF1 and UBF2 were performed with each isoform separately, it is very likely that UBF functions in dimeric form *in vivo* (26, 33, 66, 67). We, thus, cannot exclude a functional UBF1/UBF2 heterodimer involved in UBF’s assigned critical roles in rRNA transcription and this is an interesting line of further investigation.

## Experimental procedures

### Cell culture

Human HeLa and mouse NIH 3T3 and NSC34 cell lines were cultured in glutamax DMEM (Gibco/BRL) with the addition of 10% v/v fetal bovine serum (FBS) and 50 U/ml of penicillin/streptomycin. Mouse IMCD3 (IMCD) cells were cultured in DMEM/F-12 (1:1) medium with 10% v/v FBS, 2 mM glutamine, and 50 U/ml of penicillin/streptomycin. Mouse Neuro2A (N2A) cells were kept in the same conditions as NIH 3T3 but with MEMα instead of DMEM. All lines were maintained at 37 °C in 5% CO_2_. For experiments evaluating the effect of serum concentration, cells were grown in the appropriate media to 70% confluency, then incubated for 12 h in 0.5%, 10%, or 20% serum and sampled for RT-PCR or WB or FUrd incorporation.

### Generation of shUBF stably silenced clones, and silencing with siRNAs

A pLKO.1-based lentiviral shRNA vector, expressing a puromycin resistance gene and targeting the mouse *Ubf* ORF, originally from the RNAi Consortium (TRCN0000096691), was obtained from Open Biosystems (RMM3981–201808572). The shRNA sequence targets both *Ubf1* and *Ubf2* mRNAs. Lentiviral shRNA production, lentivirus particle packaging and harvesting, transductions of NIH 3T3 mouse fibroblasts, and single colony selection of clones were carried out as previously described (68, 69). Initial screening was carried out by WB, and *Ubf* expression levels further quantified by qRT-PCR. For the extra validation experiment shown in Fig. S6, a different pLKO.1-based lentiviral shRNA vector was used, targeting a sequence more towards the 5′ end of *Ubf* ORF, common to both *Ubf1* and *Ubf2* mRNAs [Fig. S6*A*; originally from the RNAi Consortium (TRCN0000096692) and obtained from Open Biosystems (RMM3981–201806555)].

siRNA-mediated silencing of *Ubf1* was carried out on NIH 3T3 cells, as a validation control, using Lipofectamine 2000 (Invitrogen) and custom-made siMAX siRNA duplexes acgaaggaggugaaggacucc/ggaguccuucaccuccuucgu (MWG) at 40 nM. Cells were sampled and analyzed by WB at 48 h post-transfection.

### SDS-PAGE and quantitative WB

Protein extraction from cells grown on 10-cm plates was carried out directly in 4X SDS-PAGE sample buffer, using a rapid extraction protocol that preserves protein phosphorylation (70). The lysates were sonicated, boiled for 5 min at 95 °C, and used immediately or stored at −20 °C. SDS-PAGE was performed using a Mini-Protean II Electrophoresis Cell (Bio-Rad) with the BlueStar prestained protein markers (MWP03; Nippon), and WB was carried out with the Mini Trans-Blot Electrophoretic Transfer Cell for wet transfer (Bio-Rad), using 48 mM Tris pH 9.2, 39 mM glycine and 20% v/v methanol as transfer buffer. Visualization of immunoreactive bands was performed with the ECL System (GE Healthcare) using ChemiDoc MP (Bio-Rad) in a series of acquisitions, selecting for signal intensities within the linear range of detection. Probing of total UBF and phospho-UBF was carried out in the same samples on blots processed in parallel, in triplicate independent experiments, to avoid any antibody cross-reactivity. For quantification of protein levels, intensity volumes (area x height) of WB signals were extracted with *ImageJ 1.49n* (NIH) and normalized using same-sample and same-membrane band intensities for the ER protein calnexin, or normalized with same-sample total protein and quantified using *ImageLab 6.0.1* software (Bio-Rad) after Ponceau S staining. Means of measurements from three independent experiments for each cell line, run in parallel, were plotted, and differences were statistically evaluated using Microsoft Excel and GraphPad Prism 8.0 (see section *Statistical Evaluation* and figure legends for details).

### RNA extraction and quantitative RT-PCR to analyze gene expression

Total RNA extraction from cell lines was carried out with the RNeasy Mini Kit (QIAGEN), including in-column DNase treatment, according to the manufacturer’s protocol. Polyadenylated RNA (polyA^+^ RNA) was affinity-purified using the Dynabeads mRNA Direct purification kit (Invitrogen). cDNA was synthesized with a random hexamer/oligo-dT primer mix, using the iScript cDNA synthesis kit (Bio-Rad).

For relative quantification of rRNAs or mRNAs of interest, qRT-PCR of cDNA (derived from total or polyA^+^ RNA, respectively) was conducted on the CFX96 Real-Time PCR system (Bio-Rad) with specific oligonucleotide primers shown in Table S1. Melting curve analysis was performed to determine the amplification specificity. Three independent experiments were conducted, and each included two no-template controls; all samples were repeated in triplicate reactions. For data normalization, the expression of *B2m* (β2-microglobulin) was used as a reference, unless otherwise stated. The Common Base method was used to allow direct quantitative comparisons across samples (71).

### Antibodies and immunofluorescence

All the primary antibodies used in this study are shown in Table S2. Primary antibodies were used in conjunction with appropriate fluorescently labeled Alexa-Fluor secondary antibodies (Thermo Fisher Scientific) for immunofluorescence, or HRP-labeled secondary antibodies (Santa Cruz and Invitrogen) for WB.

For immunofluorescence, cells were grown on coverslips, fixed with 3.7% w/v paraformaldehyde in PHEM buffer (65 mM Pipes, 30 mM Hepes, pH 6.9, 10 mM EGTA and 2 mM MgCl_2_) for 10 min and permeabilized for 15 min with 0.5% v/v Triton X-100 in PHEM. For the PoLR1A staining in Figure 7, cells were fixed/permeabilized with methanol for 10 min at −20 °C. Nuclei were counterstained with Hoechst 33342 (0.5 μg/ml). Samples were analyzed with a *Zeiss Apochromat* 63× NA 1.4 oil lens on a Zeiss *Axiovert 200M* inverted fluorescence microscope, equipped with a Zeiss *AxioCam MRm* camera.

### Growth curves and cell cycle analysis

To generate growth curves of cell lines (Fig. S1*D*), nine cultures each of NIH 3T3 WT and shUBF cells were seeded, in parallel, in twelve-well plates with 5000 cells per well. For each cell type, triplicate measurements of cell counts from each of three wells were recorded with a hemocytometer and the average of nine values was calculated at 24, 48, and 72 h after seeding.

Flow cytometric profiling of the cell cycle was conducted in 3 independent experiments with 10^5^ cells each time, fixed in 70% v/v ethanol, treated with 200 μg/ml RNAse for 45 min at 37 °C, labeled with 20 μg/ml propidium iodide, and analyzed using an *S3e Cell Sorter* (Bio-Rad) with the *FlowJo* software (Treestar). For analysis of cell cycle phases by RT-PCR or WB (Fig. S2), cells were synchronized in G1/S phase with a double thymidine block (72), washed in PBS, resuspended in fresh culture medium and collected after 2 h for enrichment in S phase. G2-arrested cells were collected after treatment with the CDK1 inhibitor RO-3306 (10 μΜ for 18 h); the mitotic (M) fraction was harvested by mechanical shake-off after nocodazole treatment (73); for G1, mitotic cells were collected after nocodazole treatment, cultured in fresh media and harvested after 6 h.

### FUrd incorporation into nascent RNA

FUrd labeling of cells was performed as described previously (74). After WT or shUBF72 cells were grown to confluency at 10% serum and then for an additional 12 h in the selected serum conditions (0.5%, 10%, and 20%; see Section *Cell Culture*), they were incubated for 15 min with fresh medium containing 2 mM 5-fluorouridine (Sigma F5130). Immunofluorescence was performed as described using an anti-BrdU primary antibody. Quantitative analysis of captured images, measuring the average mean intensity of the nucleolar signal in each cell from three independent experiments (n = 300 cells in total; 50 cells per condition), was carried out using *Imaris* (Bitplane AG, v.9.2.1).

### Quantitative Northern blots

Total RNA was extracted using Trizol (Ιnvitrogen). RNA samples were analyzed by Northern blot (75). Single-stranded DNA probes specific for mouse rRNA species were ^5’^AGAGAAAAGAGCGGAGGTTCGGGACTCCAA^3’^ (47S rRNA, as per (76)) and ^5’^GCCCAAGCATAGTTCACCATCTTT^3’^ (for 28S rRNA). Probes were non-radioactively labeled using DIG-oligonucleotide 3′-End Labeling kit (Roche). Chemiluminescence detection was performed using the DIG-luminescent detection kit (Roche) and acquired in ChemiDoc MP image system (Bio-Rad). Band intensities of chemiluminescence signals of rRNA bands were measured using *ImageJ.* Signals were expressed as band intensity per μg of total RNA loaded. Ethidium bromide-staining of rRNA bands was visualized with Syngene G:BOX (ChemiXR5).

### Quantification of signaling pathways

For assessment of components of the mTOR or RAF-MEK-ERK (MAPK) pathways, multiple cultures, seeded in parallel in an identical manner, were grown to 70% confluency in normal media with 10% FBS and then transferred for 16 h to 0.5% serum to establish the same baseline growth signaling level. Medium was then changed to either normal 10% FBS, or 10% FBS with mTOR inhibitor rapamycin at 20 mM, or 10% FBS with MAPK inhibitor U0126 at 10 mM, for 2 h and then processed for quantitative WB exactly as described in section *SDS-PAGE and quantitative WB*. At least 3 independent experiments were performed for the full set of comparisons between WT and shUBF cell lines.

### Psoralen accessibility crosslinking assay

Nuclei were enriched from cells lysed in 10 mM Tris-HCl pH 7.4, 10 mM NaCl, 3 mM MgCl_2_, 0.5% NP-40, irradiated in the presence of 4,5,8-trimethylpsoralen (psoralen) (Sigma) at 0.6 mg/ml, for 5 min on ice and repeated 4 times at 6 cm distance from the 366-nm UV light box (UVP, BlackRay model B-100A) (based on the protocol of (77)). Genomic DNA was isolated with phenol/chloroform extraction, digested with BamHI, separated on a 1.5% agarose gel, and analyzed as previously described (77, 78), using the 6.7 kb 47S rRNA gene EcoRI fragment (pMr100) (78). The ratio of “active” to “inactive” chromatin was estimated by analyzing the intensity profile of low and high mobility bands revealed by phospho-imaging on an Amersham *Typhoon* (Cytiva), using a Gaussian peak fit generated with *MagicPlotPro* (MagicPlot Systems LLC).

### Chromatin immunoprecipitation

ChIP was performed as previously described (6) with several modifications. Briefly, cells were fixed in 1% formaldehyde for 10 min and quenched with 125 mM glycine. Nuclei were isolated in swelling buffer (10 mM Tris, pH 8, 1.5 mM MgCl_2_, 10 mM KCl 0.1% Triton, 1 mM DTT), transferred in sonication buffer (50 mM Tris-HCl pH 8, 140 mM NaCl, 1 mM EDTA, 1% Triton X-100, 1% SDS and protease inhibitor cocktail), sonicated with a *Bioruptor* (Diagenode) for 30 cycles of 30 s on/30 s off at high intensity and the chromatin was diluted 10-fold in immunoprecipitation (IP) buffer (50 mM Tris-HCl pH 8, 140 mM NaCl, 1 mM EDTA, 1% Triton X-100 and protease inhibitor cocktail), followed by 1 h preclearing using Protein A sepharose beads (GE Healthcare) at RT. Each IP reaction was carried out overnight with the equivalent of 15 μg DNA in IP Buffer, at 4 °C. The antibody slurry consisted of 30 μl slurry of Protein A sepharose beads (GE Healthcare), blocked in 3 μg salmon sperm DNA and 30 μg BSA, and 5 μl antibody serum or 10 μg of Rabbit IgG per IP reaction. Beads were washed and divided into two equal parts: one was processed for ChIP (qPCR) and the other for WB analysis (“UBF ChIP” sample). For ChIP, the immunoprecipitated chromatin was eluted in elution buffer (1% SDS and 0.1 M NaHCO_3_) and reverse cross-linked using 200 mM NaCl containing 0.5 μg/μl RNase at 65  °C overnight. Samples were purified with the QIAquick PCR purification kit (QIAGEN) and analyzed in triplicate per reaction by quantitative real-time PCR on CFX96 Real-Time PCR system (Bio-Rad), with specific primers to the mouse rDNA (BK000964), as shown in Table S1. To calculate the percentage of total DNA bound, qPCR analyses of unprecipitated input samples (2% of total) were used as reference for corresponding IPs. Final enrichment was determined by subtracting the percentage of DNA of rabbit IgG controls from the corresponding UBF or Pol1 samples.

For WB analysis, beads were boiled at 95 °C for 10 min in SDS-sample buffer and separated on SDS-PAGE (“UBF ChIP” sample in Fig. 6*A1*) in parallel with non-precipitated, input samples (20% of total, per gel lane; “Input” sample in Fig. 6*A1*).

### Rescue experiments

For the rescue experiments in Fig. S5, we generated shUBF72-resistant versions of UBF1 and UBF2 harboring 3 synonymous mutations over the shRNA target sequence. The rat WT full ORFs of UBF1 and UBF2, cloned in plasmid pcDNA3.1-3xFLAG (3), were used as a template for amplification by PCR (18 cycles, annealing temperature 52 °C) with oligonucleotide pair UBFmut (Table S1). Constructs were verified with DNA sequencing (Macrogen Europe, NL). Exponentially growing WT NIH 3T3 and shUBF72 cultures in 60 mm diameter dishes were transiently transfected with 3 μg of plasmid of empty pcDNA3.1-3xFLAG plasmid or a mix of 1.5 μg pcDNA3.1-3xFLAG-3xmutUBF1 plasmid + of 1.5 μg pcDNA3.1-3xFLAG-3xmutUBF2 plasmid, by calcium/phosphate precipitation (75). Transfections were allowed to proceed for 20 h at 37 °C in 5% CO2, the transfection medium was then removed, and the dishes were cultured in fresh DMEM. Transfection efficiencies were typically in the range of 50 to 60%. Samples were retrieved for immunofluorescence, WB, or RT-PCR analysis 10h (in Fig. S5, *B2* and *B3*) or 24 h (in Fig. S5*C*) after removal of the transfection medium.

### Statistical evaluation of quantitative analysis

To evaluate statistical significance of differences between comparisons of values expressed as means ± SD of at least three different independent experiments run in parallel, the following statistical analysis was employed, as appropriate: Welch’s *t* test for comparisons between two groups, not assuming equal variance; one-way ANOVA with Dunnett’s correction or two-way ANOVA with Tukey’s correction for differences of one or two variables, respectively, among more than two groups. Growth curve and cell cycle differences (Fig. S1) were compared by repeated measures two-way ANOVA for analysis of parameter means with Sidak post hoc test for multiple comparisons. If other statistical analyses were employed, they are mentioned in the figure legend. A *p* value＜0.05 was regarded statistically significant (∗), <0.01 very significant (∗∗), <0.001 highly significant (∗∗∗), and <0.0001 extremely significant (∗∗∗∗). Statistical analyses were performed using the GraphPad Prism 8.0 Software (GraphPad, Inc).

## Data availability

The manuscript contains all data described within the text.

## Websites

W1: https://www.phosphosite.org/proteinAction.action?id=1131&showAllSites=true.

W2: https://www.genecards.org/cgi-bin/carddisp.pl?gene=POLR1A.

W3: https://www.genecards.org/cgi-bin/carddisp.pl?gene=POLR1E.

W4: https://www.proteinatlas.org/ENSG00000068654-POLR1A.

W5: https://www.proteinatlas.org/ENSG00000137054-POLR1E.

W6: http://www.signalingpathways.org/ominer/query.jsf?geneSearchType=gene&findMax=y&gene=POLR1A&foldChangeMin=2&foldChangeMax=30&significance=0.05&species=all&reportsBy=pathways&omicsCategory=tm&countMax=3000.

W7: http://www.signalingpathways.org/ominer/query.jsf?geneSearchType=gene&findMax=y&gene=POLR1E&foldChangeMin=2&foldChangeMax=30&significance=0.05&species=all&reportsBy=pathways&omicsCategory=tm&countMax=3000.

## Supporting information

This article contains supporting information (2, 30, 31, 79).

## Conflict of interest

The authors declare that they have no known competing financial interests or personal relationships that could have appeared to influence the work reported in this paper.
