# Supplementary material for: Transcription factor UBF depletion in mouse cells results in downregulation of both downstream and upstream elements of the rRNA transcription network
Source: J Biol Chem. 2023 Sep 1;299(10):105203. doi: 10.1016/j.jbc.2023.105203 (PMC10558777; doi:10.1016/j.jbc.2023.105203)
Supplement: Supplemental data [file mmc1.pdf]

SUPPORTING INFORMATION

Figures S1-S6

Tables S1-S2

Supplementary Figure Legends

Transcription factor UBF depletion in mouse cells results in downregulation of both downstream and upstream elements of the rRNA transcription network

Andria Theophanous[1], Andri Christodoulou[1], Charalambia Mattheou[1], Dany S. Sibai[2,3], Tom Moss[2,3], and Niovi Santama[1*]

[1]Department of Biological Sciences, University of Cyprus, Nicosia, Cyprus

[2]Laboratory of Growth and Development, St-Patrick Research Group in Basic Oncology, Cancer Division of the Quebec University Hospital Research Centre, Quebec, Canada

[3] Department of Molecular Biology, Medical Biochemistry and Pathology, Faculty of Medicine, Laval University, Quebec, Canada.

*Address correspondence to Niovi Santama

Department of Biological Sciences, University of Cyprus, University Avenue 1,

1678 Nicosia, Cyprus

(Tel. +357-22-892881, e-mail: santama@ucy.ac.cy)

Short title:

Concerted regulation of rRNA transcription on UBF depletion

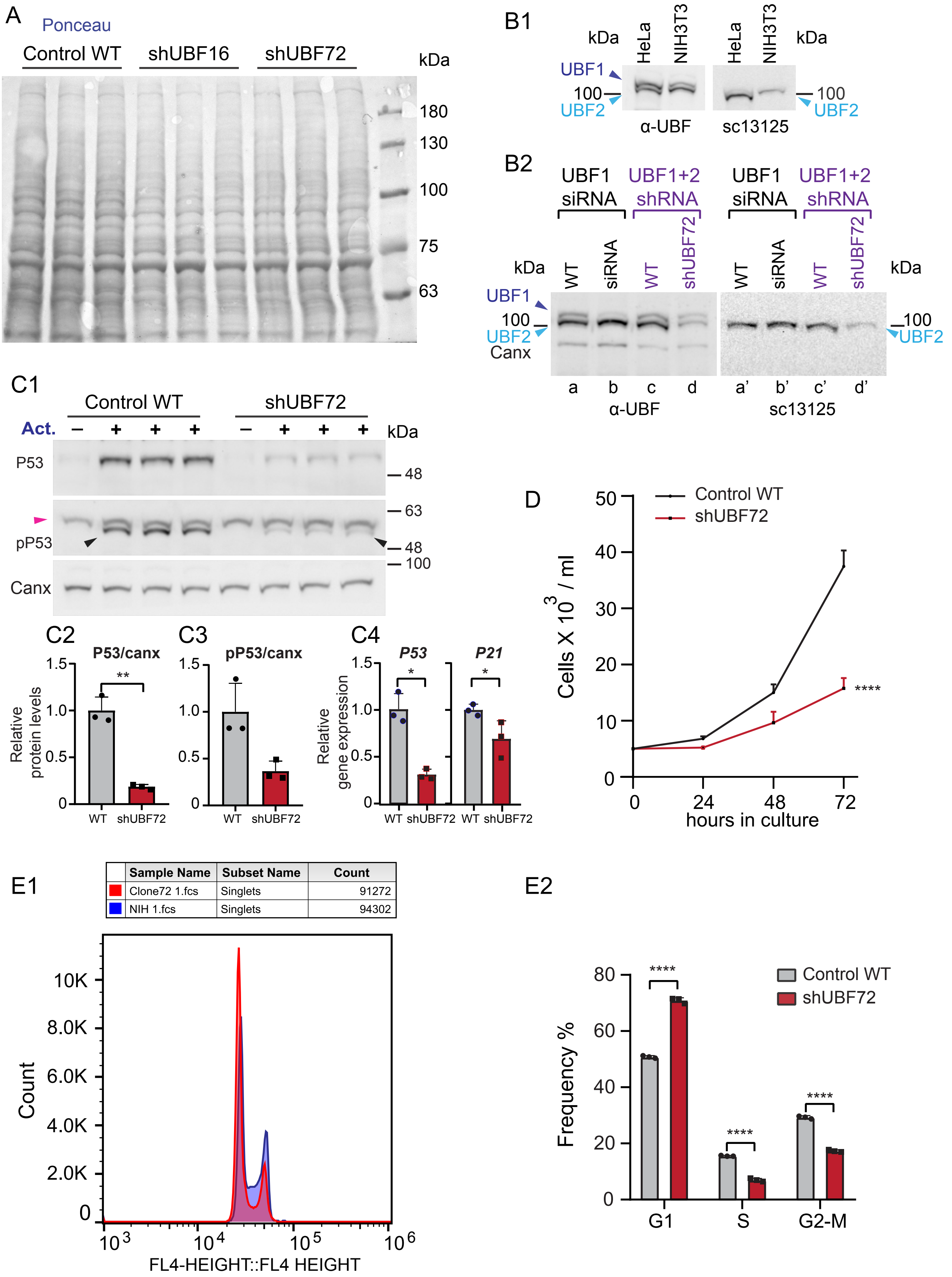
A
Ponceau
Control WT
shUBF16
shUBF72
kDa
180
130
100
75
63
B1
kDa
HeLa
NIH3T3
UBF1
100
UBF2
α-UBF
sc13125
B2
UBF1 siRNA
UBF1+2 shRNA
WT
siRNA
shUBF72
Canx
a
b
c
d
a'
b'
c'
d'
C1
Act.
P53
pP53
48
63
Canx
C2
P53/canx
Relative protein levels
C3
pP53/canx
C4
P53
P21
Relative gene expression
D
Cells X 10 3 / ml
hours in culture
****
E1
Sample Name
Subset Name
Count
Clone72 1.fcs
Singlets
91272
NIH 1.fcs
Singlets
94302
Count
FL4-HEIGHT::FL4 HEIGHT
E2
Frequency %
G1
S
G2-M

FIGURE S1

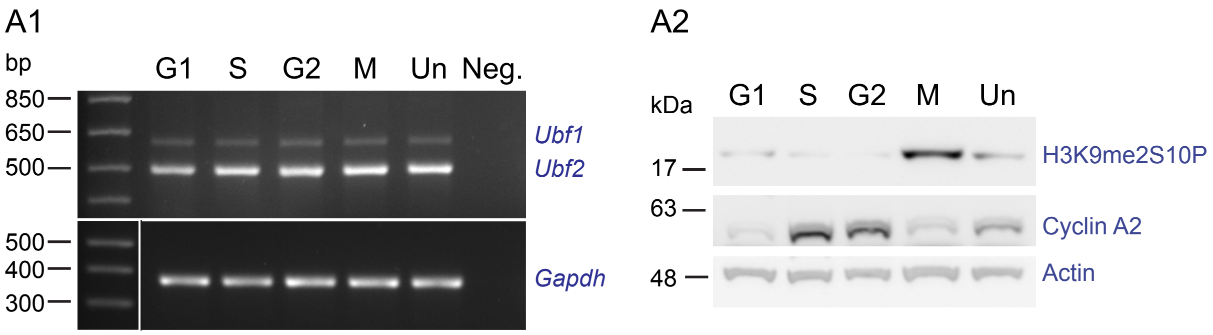
A1
bp
G1 S G2 M Un Neg.
850
650
500
Ubf1
Ubf2
500
400
300
Gapdh
A2
kDa
G1 S G2 M Un
17
H3K9me2S10P
63
Cyclin A2
48
Actin

FIGURE S2

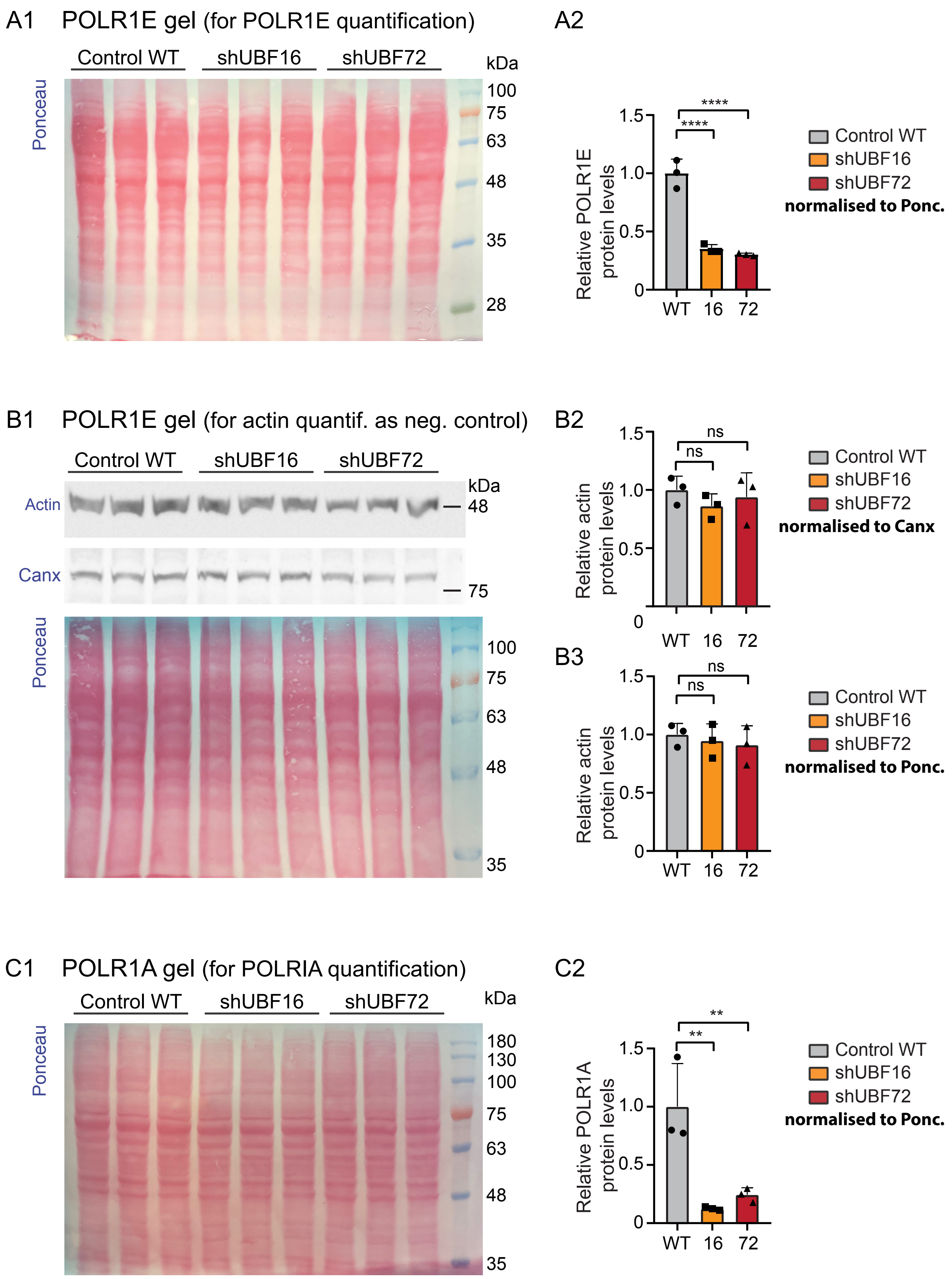

A1 POLR1E gel (for POLR1E quantification)
Control WT
shUBF16
shUBF72
kDa
Ponceau
100
75
63
48
35
28
A2
Relative POLR1E protein levels
1.5
1.0
0.5
0
****
****
WT
16
72
Control WT
shUBF16
shUBF72
normalised to Ponc.
B1 POLR1E gel (for actin quantif. as neg. control)
Control WT
shUBF16
shUBF72
Actin
Canx
Ponceau
kDa
48
75
100
75
63
48
35
B2
Relative actin protein levels
ns
ns
Control WT
shUBF16
shUBF72
normalised to Canx
B3
Relative actin protein levels
ns
ns
Control WT
shUBF16
shUBF72
normalised to Ponc.
C1 POLR1A gel (for POLRIA quantification)
Control WT
shUBF16
shUBF72
kDa
Ponceau
180
130
100
75
63
48
35
C2
Relative POLR1A protein levels
**
**
Control WT
shUBF16
shUBF72
normalised to Ponc.

FIGURE S3

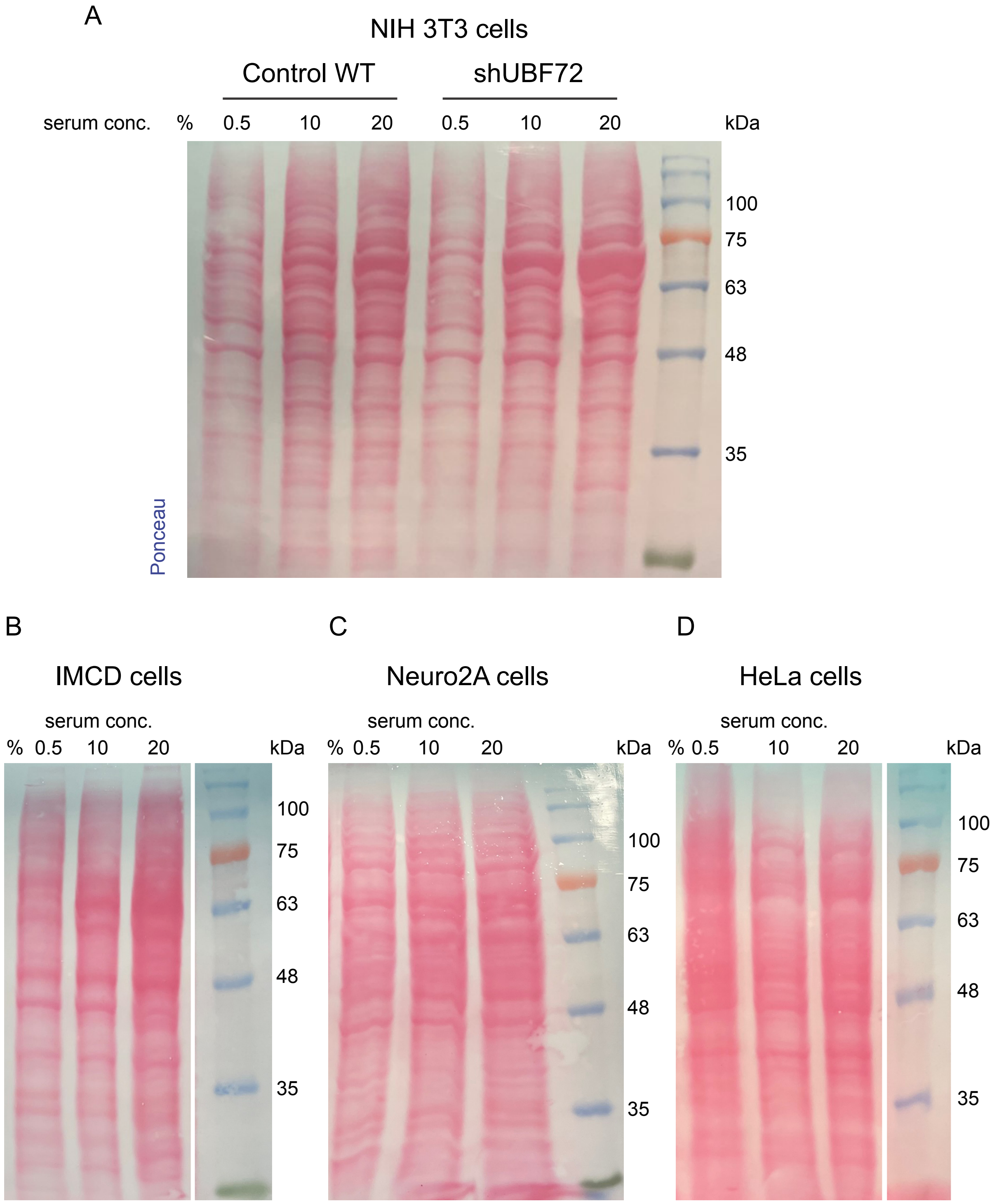
A
NIH 3T3 cells
Control WT
shUBF72
serum conc. % 0.5 10 20 0.5 10 20
kDa
100
75
63
48
35
Ponceau
B
IMCD cells
serum conc.
% 0.5 10 20
kDa
100
75
63
48
35
C
Neuro2A cells
serum conc.
% 0.5 10 20
kDa
100
75
63
48
35
D
HeLa cells
serum conc.
% 0.5 10 20
kDa
100
75
63
48
35

FIGURE S4

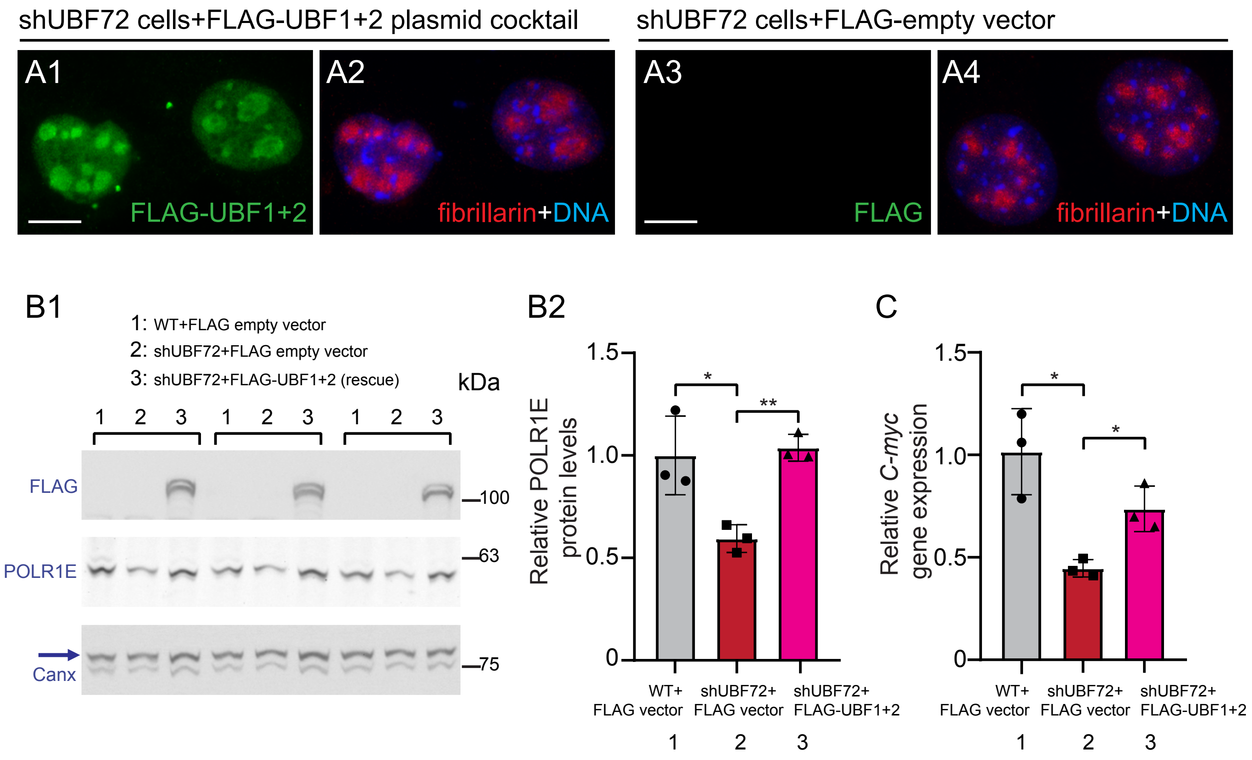
shUBF72 cells+FLAG-UBF1+2 plasmid cocktail
shUBF72 cells+FLAG-empty vector
A1
FLAG-UBF1+2
A2
fibrillarin+DNA
A3
FLAG
A4
fibrillarin+DNA
B1
1: WT+FLAG empty vector
2: shUBF72+FLAG empty vector
3: shUBF72+FLAG-UBF1+2 (rescue)
kDa
1 2 3 1 2 3 1 2 3
FLAG
POLR1E
Canx
100
63
75
B2
Relative POLR1E protein levels
1.5
1.0
0.5
0
*
**
WT+ FLAG vector
shUBF72+ FLAG vector
shUBF72+ FLAG-UBF1+2
1 2 3
C
Relative C-myc gene expression
1.5
1.0
0.5
0
*
*
WT+ FLAG vector
shUBF72+ FLAG vector
shUBF72+ FLAG-UBF1+2
1 2 3

FIGURE S5

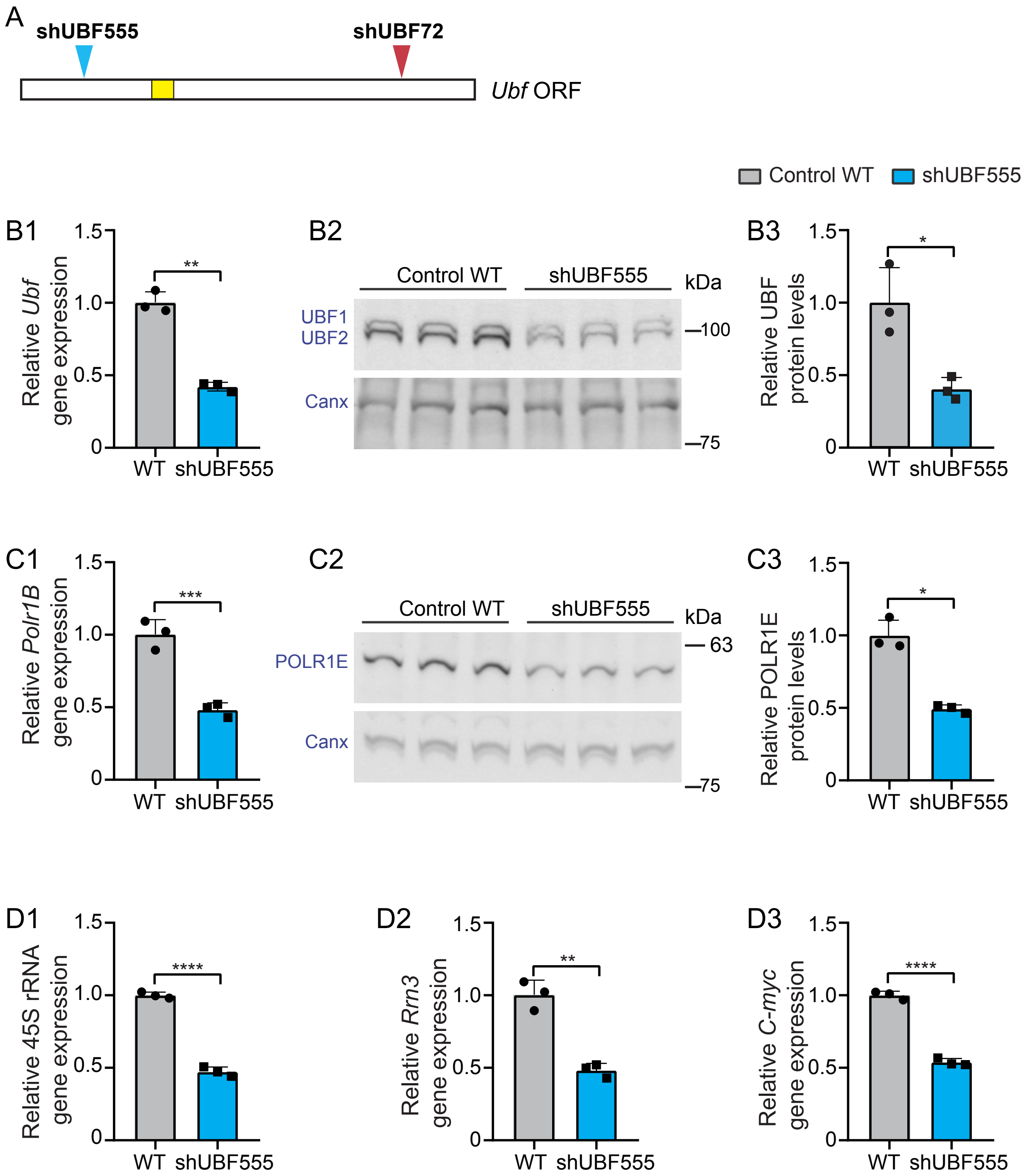
A
shUBF555
shUBF72
Ubf ORF
Control WT
shUBF555
B1
Relative Ubf gene expression
**
WT
shUBF555
B2
Control WT
shUBF555
kDa
UBF1
UBF2
Canx
100
75
B3
Relative UBF protein levels
*
C1
Relative Polr1B gene expression
***
C2
POLR1E
63
C3
Relative POLR1E protein levels
D1
Relative 45S rRNA gene expression
****
D2
Relative Rrn3 gene expression
D3
Relative C-myc gene expression

FIGURE S6

Gene target (mouse)	Primer Sequence (5'-3')
UBF	Forward: GCCGGATGCCACTACGAAG (19)<br>Reverse: CCAGGGCCTTATGAATCCATTTC (23)<br>For RT-qPCR, amplifying partial sequences of both *Ubf1+Ubf2* (Fig.1, 5).
UBFiso	Forward: CCAGAGGGAGAAACAGGAGTTCG (23)<br>Reverse: TCTTCCTCCGGCAAGCTCTCAAG (23)<br>For RT-PCR, generating different sized-amplicons for *Ubf1* and *Ubf2*, that can be resolved by agarose electrophoresis (Fig.S2).
UBFmut	Forward: GACCGCGC**G**GCATA**T**AAAGA**G**TACATC (27)<br>Reverse: GATGTA**C**TCTTT**A**TATGC**C**GCGCGGTC (27)<br>For RT-PCR using WT plasmid templates, to generate, shUBF-resistant plasmid versions containing rat UBF1 or UBF2 that each carry 3 synonymous mutations (underlined) within the shUBF72 target sequence (for rescue experiments in Fig.S5).
ETS_5-113<br>(47S pre-rRNA)	Forward: ACACGCTGTCCTTTCCCTAT (20)<br>Reverse: CGACAGACCCAAGCCAGTAA (20)
45S<br>(45S pre-rRNA)	Forward: CTCTTAGATCGATGTGGTGCTC (22)<br>Reverse: GCCCGCTGGCAGAACGAGAAG (21)
RNA Pol I subunit B<br>POLR1B (RPA135)	Forward: CAGGCCATACCTCCCTTTGAA (21)<br>Reverse: TCCCTCGGTAGGTGCTCTTTC (21)
Cyclin D	Forward: GCGTACCCTGACACCAATCTC (21)<br>Reverse: CTCCTCTTCGCACTTCTGCTC (21)
TAF1A	Forward: GGCTGTGGCAGAGGATAACC (20)<br>Reverse: GCACTTGTCGTCTGAGCAAAATC (23)
TAF1B	Forward: CTTCCTGCCACAATGTCACAG (21)<br>Reverse: CCTTTCTCATGCTTGCTTCTCTG (23)
TAF1C	Forward: CGGTCCACTTGGCATGACT (19)<br>Reverse: GGCAAAGGACAAGGTCGGA (19)
TBP	Forward: GCTCTGGAATTGTACCGCAG (20)<br>Reverse: TGACTGCAGCAAATCGCTTG (20)
RRN3	Forward: GCCCTGGTTGAATAGAAGTCAG (22)<br>Reverse: AGACATGGTCTAAGGAAGACAGT (23)
CTCF	Forward: GATCCTACCCTTCTCCAGATGAA (23)<br>Reverse: GTACCGTCACAGGAACAGGT (20)
TTF1	Forward: TGGAGAACCTGCTAGAGACTTC (22)<br>Reverse: TGTTTCACGCACTTTTGCTGA (21)
C-MYC	Forward: GCGACTCTGAAGAAGAGCAAG (21)<br>Reverse: GCCTCGGGATGGAGATGAG (19)

P53	Forward: GTATTTCACCCTCAAGATCC (20)<br>Reverse: TGGGCATCCTTTAACTCTA (19)
P21	Forward: CGAGAACGGTGGAACTTTGAC (21)<br>Reverse: CAGGGCTCAGGTAGACCTTG (20)
B2M	Forward: TGCTACTCGGCGCTTCAGTC (20)<br>Reverse: AGGCGGGTGGAACTGTGTTAC (21)
PUMI	Forward: TGAGGTGTGCACCATGAAC (19)<br>Reverse: CAGAATGTGCTTGCCATAGG (20)
GAPDH	Forward: CTTCATTGACCTCAACTACATGGT (24)<br>Reverse: TCATGGATGACCTTGGCCAGGG (22)
IGS3 (rDNA)	Forward: GCAGCGAGCACCAGAAAACAAC (22)<br>Reverse: GACTCGGGCACCCAAAAACGAA (22)
SpPr (rDNA)	Forward: AGTGGTGACAAGTTTCGGGAACG (23)<br>Reverse: ACACCATCTCCGAGACGCTCC (21)
Tsp (rDNA)	Forward: AGGACGGTCTCTAACAAGGAGG (22)<br>Reverse: GACAAGAGAGGGCTTCTGGAGG (22)
To/Pr (rDNA)	Forward: AGTTGTTCCTTTGAGGTCCGGT (22)<br>Reverse: CAGCCTTAAATCGAAAGGGTCT (22)
28S (rDNA)	Forward: CTCCCGACGTACGCAGTTTTATCC (24)<br>Reverse: ATCGTTTCGGCCCCAAGACC (20)

TableS1 List of oligonucleotide primer pairs used for qRT-PCR analysis.

Antigen	Source/Reference	Dilution	Source
P70 (S6K)	Cell Signalling 9202S	1:1000 (WB)	Rabbit
Phospho-P70 (S6K) (Thr 389)	Cell Signalling 9205S	1:500 (WB)	Rabbit
EIF4E	Cell Signalling 9742S	1:1000 (WB)	Rabbit
Phospho-EIF4E (Ser 209)	Cell Signalling 9741S	1:500 (WB)	Rabbit
RB	Cell Signalling 9313S	1:1000 (WB)	Rabbit
Phospho-RB (Ser 780)	Cell Signalling 8180S	1:500 (WB)	Rabbit
UBF	Santa Cruz (F-9) sc-13125	1:1000 (WB) 1:200 (IF)	Mouse
Phospho-UBF (Ser 484)	Abcam ab182583	1:1500 (WB)	Rabbit
Calnexin	Santa Cruz sc-11397	1:500 (WB)	Rabbit
POLR1A (RPA194)	Santa Cruz sc-48385	1:500 (WB) 1:50 (IF)	Mouse
POLR1E (RPA49)	Santa Cruz sc-398270	1:200 (WB)	Mouse
Actin	Santa Cruz sc-5286	1:500 (WB)	Mouse
Fibrillarin	Cell Signalling 2639S	1:400 (IF)	Rabbit
BrdU	Abcam ab 6326	1:250 (IF)	Rat
P53	Proteintech 60283-2-Ig	1:500 (WB)	Mouse mab
Phospho-P53 (Ser 392)	Proteintech 28963-1-AP	1:500 (WB)	Rabbit
dimethyl (Lys9) phospho (Ser10) histone H3	Millipore #05-1354	1:1000 (WB)	Mouse mab
Cyclin A2	Abcam Ab38 (E23.1)	1:500 (WB)	Mouse mb
RRN3	Moss Laboratory (ref. 2)	1:500 (WB)	Rabbit
UBF	Moss Laboratory (ref. 79)	1:1000 (WB) 1:200 ChIP	Rabbit
POLR1A (RPA194)	Moss Laboratory (ref. 79)	1:200 ChIP	Rabbit
FLAG epitope	Sigma F1804	1:2000 (WB) 1:500 (IF)	Mouse mab

Table S2

List of primary antibodies used for immunofluorescence (IF), WB, and ChIP.

SUPPLEMENTARY FIGURE LEGENDS

Figure S1

UBF silencing validation, growth curves and cell cycle analysis of WT and shUBF72 cells

(A) Full WB membrane of the experiment shown in Fig.1 A1, here stained with Ponceau.

(B1) Validation of antibody specificity via comparison of UBF immunostaining by WB with total extracts from HeLa human cell line or NIH 3T3 mouse cell line, using a custom house antibody (79) (left panel) and a commercially available one (sc13125; Santa Cruz; right panel). Two bands, consistent to UBF1 and UBF2 (30, 31), are detected in both HeLa and NIH 3T3 cells with the house anti-UBF, while only the lower band (corresponding to UBF2) is detectable with sc13125 antibody.

(B2) Evaluation of UBF silencing with detection of a reduced UBF1 band (upper band), following *Ubf1*-specific siRNA silencing at 48h post-transfection (compare lanes a+b) but no effect on the UBF2 band (lower band, compare lanes a+b and also a'+b'). In contrast, in the shUBF72 clone, in which both *Ubf1* and *Ubf2* are targeted, a clear reduction of both upper (UBF1) and lower (UBF2) bands is visible (compare lanes c+d and also c'+d'). In all comparisons, a WT-sample of equal loading is displayed along the silenced sample. Calnexin detection in the samples is shown to confirm equal loading within each set of paired samples.

(C1-C3) Representative WB analysis (C1) and quantitation in 3 independent experiments of P53 protein levels (C2) and phospho-P53 (Ser 392) (C3) in resting conditions (Act-) or after induction of nucleolar stress by actinomycin treatment (0.5 μg/ml for 2 h; Act+) of WT NIH 3T3 cells (blue) and clone shUBF72 (red), indicates relative reduction of normalized average values of both markers in shUBF72. In the pP53 WB panel (C3), the pertinent band is indicated with black arrowheads and an unrelated non-actinomycin responsive unspecific band (serving as internal control) is shown with a pink arrowhead.
Statistical significance of differences was assessed by Welch's t-test**.**

(C4) Quantification of relative gene expression of *P53* and *P21*, as markers of nucleolar stress, by qRT-PCR comparing WT and shUBF72 cells, grown in parallel, in 3 independent experiments, shows reduction of *P53* gene expression, comparable to reduction of P53 protein levels shown in (C2) and moderate decline of *P21*.
Statistical significance of differences was assessed by Welch's t-test**.**

(D) Growth curves of WT NIH 3T3 cells (blue) and clone shUBF72 (red) reveal a greatly reduced growth rate in UBF-silenced cells. Shown are the average values ± SD of triplicate measurements in each of three replicate cultures per cell type, seeded with 5000 cells/well, grown in parallel for 72 h and sampled every 24 h.

(E1, E2) Cell cycle profile by flow cytometry, with a representative cell cycle profile of WT NIH 3T3 cells (blue) and clone shUBF72 (red) in one of 3 independent experiments, each counting 10^5

cells (E1). Quantification with average values in the three experiments (E2) reveals an increased fraction of the cell population in G1, specifically in shUBF72 compared with WT (70.93 ± 0.9% in shUBF72 vs. 50.83 ± 0.45% in WT), and a concomitant reduction in phases S (7.04 ± 0.55% in shUBF72 vs. 15.63 ± 0.15% in WT) and G2/M (17.37 ± 0.3% in shUBF72 vs. 29.37 ± 0.5% in WT).

The differences in D and E2 are statistically significant as assessed by stacked two-way ANOVA for analysis of parameter means with Sidak post hoc test for multiple comparisons.

Figure S2

Cell cycle analysis of UBF isoform gene expression

(A1) Semi-quantitative RT-PCR analysis of *Ubf* expression in WT NIH 3T3 cells with oligonucleotide primers bracketing the alternatively spliced sequence within the *Ubf* pre-mRNA (Table S1), resulted in the amplification of a diagnostic amplicon of 612 bp for *Ubf1* and of 501 bp for *Ubf2* isoforms. Shown are samples that were enriched in cell cycle phases G1, S, G2, and M (prepared from synchronized cultures as described in *Experimental Procedures*), an unsynchronized sample (Un), or a negative control reaction (N) (top panel). Equivalent reactions from the same samples with expression of housekeeping gene *Gapdh* are shown in parallel as loading controls (bottom panel).

(A2) WB of equivalent samples as in A1, probed with cell cycle-specific markers to illustrate the effectiveness of synchronization protocols employed: mitotic marker dimethyl (Lys9) phospho (Ser10) histone H3 (H3K9me2S10P) (top panel), and Cyclin A2, essential for the control of the cell cycle at the G1/S and G2/M transitions (middle panel). Actin labeling is shown as loading control (bottom panel).

Figure S3

Supplementary analysis related to Fig.7

(A1) Full WB membrane of the experiment shown in Fig.7 A1, here stained with Ponceau.

(A2) Alternative method of POLR1E relative protein level quantification to the method shown in Fig.7 A2, here employing sample normalization using the total, same-sample Ponceau signal. Statistically significant large differences in POLR1E protein levels are detected in both shUBF16 and 72, relative to the WT cells.

(B1) WB analysis (actin in top panel, calnexin in middle panel) of the same experimental set as in Fig.7 A1 and full WB membrane of the experiment stained with Ponceau (lower panel), comparing WT, shUBF16 and shUBF72 cells.

(B2, B3) Corresponding quantification of protein levels for actin, relative to WT, with sample normalization using same-sample calnexin signals (B2) or using total protein content as visualized by Ponceau staining (B3). Both quantifications reveal that actin (used as negative

control in this experiment) remains essentially unaltered in all cell types and unaffected by UBF silencing with statistically insignificant small fluctuations.

(C1, C2) Full WB membrane of the experiment shown in Fig.7 B1, here stained with Ponceau. Alternative method of POLR1A relative protein level quantification to the method shown in Fig.7 B1, here employing same-sample Ponceau signal for normalization. Similar to the results obtained for POLR1E, statistically significant large differences in POLR1A protein levels are detected in both shUBF16 and 72, relative to the WT cells.

All statistical evaluations were performed with one-way ANOVA with Dunnett's method.

Figure S4

Supplementary analysis related to Fig.8

(A) Ponceau-stained full-size WB membrane of the experiment depicted in Fig.8 B1 and used for sample normalization in the quantification shown in Fig.8 B2.

(B-D) Ponceau-stained full-size WB membranes of the experiments depicted in Fig.8 D (mouse IMCD cells, panel B), in Fig.8 E (mouse Neuro2A cells, panel C) and in Fig.8 F (human HeLa cells, panel D) and used, respectively, for sample normalization in the quantifications shown in Fig.8 B-D.

Figure S5

Rescue of RNA Pol1 and *C-myc* by UBF1+UBF2 cocktail expression

(A1-A4) Confirmation by immunofluorescence of correct nucleolar localization in shUBF72 cells transiently transfected with a plasmid cocktail expressing shUBF72 oligo-resistant, synonymous triple mutant FLAG-tagged rat UBF1 and UBF2 in rescue experiments [FLAG in green (A1), fibrillarin (red) and DNA (blue) overlay in the nucleolus (A2)]. FLAG immunolabeling cannot discriminate between the two isoforms, but FLAG-UBF1 and FLAG-UBF2 individually also localise to the nucleolus (data not shown). Labeling was carried out in parallel in shUBF cells transiently transfected with the empty FLAG vector (A3; fibrillarin and DNA overlay in A4). Scale bars 10 μm.

(B1-B2) Comparison by WB of POLR1E protein levels (middle panel) in WT of 3 independent rescue experiments (B1) and its quantification (B2). Samples from WT (1) and shUBF72 cells (2) transfected with a FLAG empty expression vector were compared with shUBF72 cells transfected with a cocktail mix of the same vector expressing silencing oligo-resistant FLAG-tagged rat UBF1 and UBF2 (double bands in 3). Top panel in B1 confirms expression of FLAG-UBF1+UBF2 and bottom panel displays corresponding calnexin levels used for normalization. Expression of FLAG-UBF1+2 results in full restoration of POLR1E protein levels. Samples were harvested 10 h after removal of the transfection medium. Statistical significance of differences between pairs was assessed by Welch's t-test.

(C) Examination of *C-myc* expression in rescue experiments from 3 independent experiments, in a same set up as in B, reveals partial significant increase of *C-myc* mRNA levels in shUBF72 cells, specifically in the presence of the FLAG-UBF1+2 cocktail, compared to the presence of the empty FLAG vector. Samples were retrieved 24 h after removal of the transfection medium. Statistical significance of differences between pairs was assessed by Welch's t-test.

Figure S6

Further validation of UBF silencing phenotypes in new shUBF555 clone, expressing a different shUBF vector

(A) Sketch illustrating the localization of the target sequence of shUBF555 silencing oligos (blue arrowhead) in comparison with shUBF72/shUBF16 (red arrowhead) within the *Ubf* ORF. The yellow highlight denotes the sequence that is unique to UBF1 and lacking in UBF2.

(B1) Quantification of relative *Ubf* mRNA levels by qRT-PCR, depicting the normalized average knockdown in shUBF555 cells, expressed as a fraction of the normalized average WT control values. Error bars correspond to SD of 3 independent experiments. Depletion of *Ubf* in shUBF555 is comparable to that observed in shUBF72 and 16.

(B2) Comparison by WB of UBF protein levels (UBF1 and UBF2) in WT and shUBF555 (upper panel) and calnexin levels for normalization (lower panel) in samples from 3 independent experiments.

(B3) Corresponding quantification of the 3 independent experiments shown in B2, displaying the average value of total UBF protein levels normalized to calnexin and expressed relative to the WT control value (set at 1) ± standard deviation (SD). The marked reduction of UBF protein levels in shUBF555 is below 50% of WT levels and consistent with mRNA reduction in B1.

(C1) Quantification of relative *Polr1B* mRNA levels by qRT-PCR, depicting the significant normalized average knockdown in shUBF555 cells.

(C2-C3) WB analysis in 3 independent experiments (C2) and corresponding quantification (C3) of average protein levels of POLR1E protein levels, normalized to same-lane calnexin, reveals significant reduction of RNA Pol1 subunit in shUBF555, relative to WT samples.

(D1-D3) Quantification in 3 independent experiments of 45S rRNA levels (D1) and mRNA levels of PIC factor *RRN3* (D2) and transcription factor *C-myc* (D3), comparing shUBF555 and WT cells, confirms reductions in all cases, similar to those reported for shUBF72.

Statistical significance of differences in all panels was assessed by Welch's t-test.

SUPPLEMENTARY TABLE LEGENDS

Table S1

List of oligonucleotide primer pairs used for qRT-PCR analysis.

Table S2

List of primary antibodies used for immunofluorescence, WB and ChIP.
